# *N*MR^2^-Based Drug Discovery Pipeline Presented on the Oncogenic Protein KRAS

**DOI:** 10.1021/jacs.4c16762

**Published:** 2025-04-14

**Authors:** Matthias Bütikofer, Felix Torres, Harindranath Kadavath, Nina Gämperli, Marie Jose Abi Saad, Daniel Zindel, Nicolas Coudevylle, Roland Riek, Julien Orts

**Affiliations:** a Department of Pharmaceutical Sciences, University of Vienna, Josef-Holaubek-Platz 2, 2F 353, Vienna, A-1090, Austria; b Institute for Molecular Physical Science, Vladimir Prelog Weg 2, Zürich, 8093, Switzerland; c NexMR AG, Wiesenstrasse 10A, Schlieren, 8952, Switzerland; d St. Jude Children’s Research Hospital, 262 Danny Thomas Place, Memphis, Tennessee, 38105, United States

## Abstract

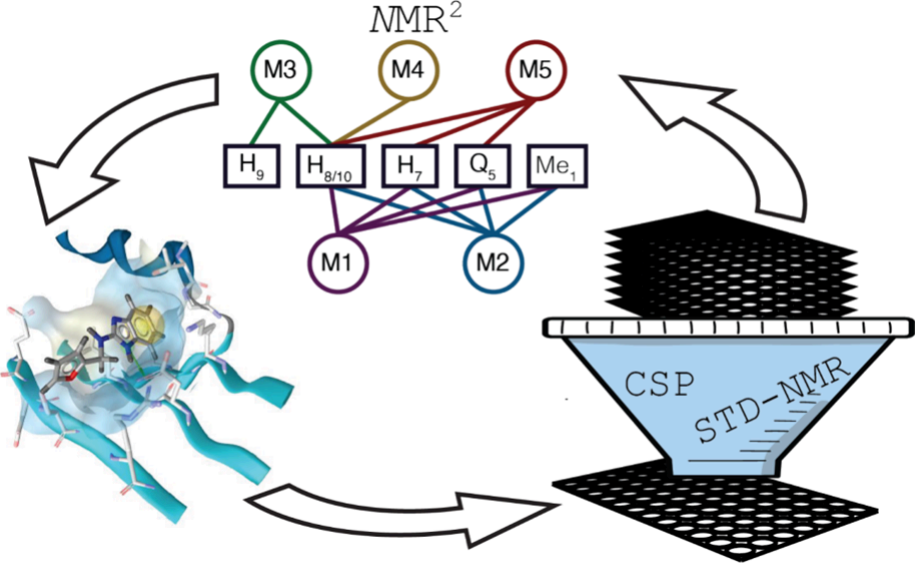

Fragment-based drug discovery has emerged as a powerful approach for developing therapeutics against challenging targets, including the GTPase KRAS. Here, we report an NMR-based screening campaign employing state-of-the-art techniques to evaluate a library of 890 fragments against the oncogenic KRAS G12V mutant bound to GMP-PNP. Further HSQC titration experiments identified hits with low millimolar affinities binding within the SI/SII switch region, which forms the binding interface for the effector proteins. To elucidate the binding modes, we applied NMR molecular replacement (*N*MR^2^) structure calculations, bypassing the need for a conventional protein resonance assignment. Traditionally, *N*MR^2^ relies on isotope-filtered nuclear Overhauser effect spectroscopy experiments requiring double-labeled [^13^C,^15^N]-protein. We introduce a cost-efficient alternative using a relaxation-based filter that eliminates isotope labeling while preserving structural accuracy. Validation against standard isotopically labeled workflows confirmed the equivalence of the derived protein–ligand structures. This approach enabled the determination of 12 *N*MR^2^ KRAS–fragment complex structures, providing critical insights into structure–activity relationships to guide ligand optimization. These results demonstrate the streamlined integration of *N*MR^2^ into a fragment-based drug discovery pipeline composed of screening, binding characterization, and rapid structural elucidation with or without isotopic labeling.

## Introduction

Developing novel therapeutic strategies against challenging oncogenic targets remains a central pursuit in medicinal chemistry. Among these, the protein K-Ras-4B (KRAS) was considered a particularly elusive target and today remains an active field of research. KRAS is one of the four RAS proteins, belonging to the family of small GTPases.^1−4^ It acts as a switch in cell signaling and binds GDP or GTP in an inactive or active state, which triggers conformational changes in the switch I and II regions, regulating the interaction with downstream effectors.^5^ The hydrolysis of GTP inactivates KRAS, a process catalyzed by GTPase-activating proteins.^6^ However, the single-point mutation of G12, G13, or Q61 attenuates the hydrolysis reaction.^7^

KRAS was seen as an undruggable target for a long time because it lacks a clear binding pocket. However, several potential drugs have reached clinical trials and the market in recent years.^8^ Most of those approaches were driven by fragment-based drug discovery (FBDD).^9^ A shallow binding pocket between the switch I and switch II regions of KRAS has been identified, and nanomolar binders for this pocket were developed.^10−13^ Furthermore, covalent inhibitors targeting the G12C mutant by binding to the “switch II pocket” have been developed.^14,15^ Based on the structure–activity relationship (SAR) obtained with the covalent binders, potent molecules have been designed that specifically target the G12D mutant by forming a salt bridge with the D12 residue.^16^

Nuclear magnet resonance (NMR) is a highly favorable technique for FBDD as it can detect weakly interacting molecules with an unmodified target in solution.^17^ Furthermore, it offers a unique advantage in the study of protein–ligand interactions, as it provides both structural and dynamic information at atomic resolution.^18^ However, traditional NMR techniques often require prior protein assignment, which can be time-consuming in measurement time and analysis and challenging. Additionally, ^13^C labeling of the target is required, which is expensive and not always possible. Therefore, X-ray crystallography is most often the method of choice to establish a structure–activity relationship in an FBDD campaign. However, the crystallization process is a bottleneck and potentially promising drug discovery projects are aborted when a target does not show good crystallization properties.^19,20^

NMR molecular replacement (*N*MR^2^) is a recently established method that enables ligand–protein complex structure determination without prior protein assignment. It relies on nuclear Overhauser enhancement (NOE) cross-peaks between ligand hydrogens and methyl groups of the protein, which can be easily obtained by protein-filtered 2D NOESY (nuclear Overhauser effect spectroscopy) experiments. The method has been demonstrated to work on peptides, drug-like molecules, and fragments, showing its potential in FBDD campaigns to leverage projects that are not accessible with X-ray crystallography.^21−23^

**Figure 1:**
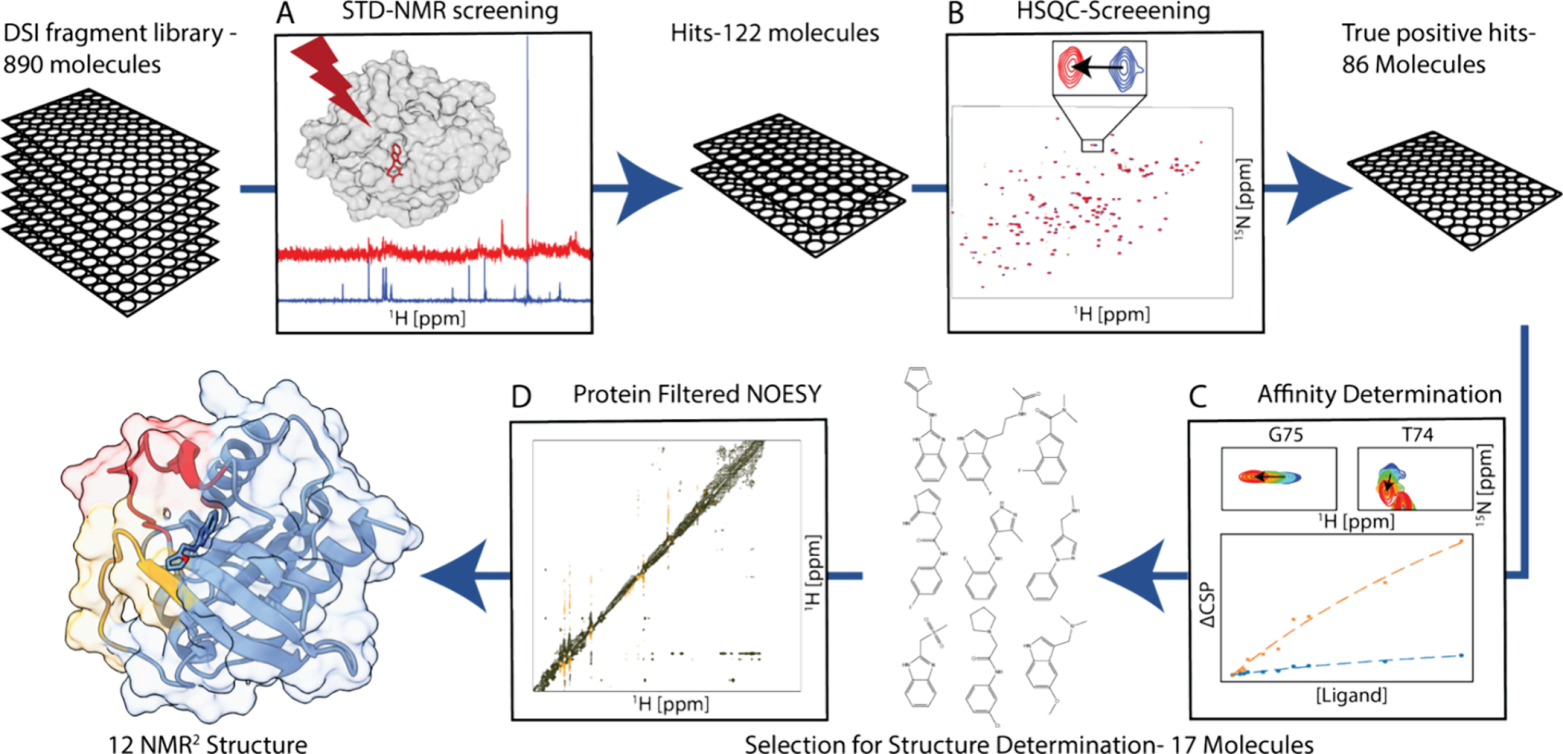
NMR-based drug discovery pipeline against oncogenic protein KRAS G12V. (A) The 890 molecules of the DSI-poised fragment library are screened in mixtures of six compounds against KRAS G12V in a GMP-PNP bound state. An example STD-NMR spectrum is shown in red, and its 1D reference is shown in blue. (B) Screening of the primary hits with [^15^N,^1^H]-HSQC NMR with the apo KRAS spectrum in blue and in the presence of a ligand in red. The shift of residue G75 upon binding is shown in a zoomed-in view. (C) The affinity of the molecules showing the most promising chemical shift perturbation is determined by ligand-titrated [^15^N,^1^H]-HSQC NMR. (D) By measuring a protein-filtered [^1^H,^1^H]-NOESY spectrum for the most promising fragments, a protein–fragment complex structure is calculated using *N*MR^2^ to establish a structure–activity relationship, illustrated with the complex structure of fragment **1** bound to KRAS, for which the switch I and II regions are highlighted in orange and red.

In this work, we present how we use *N*MR^2^ in an FBDD pipeline based on NMR to establish a SAR for the switch I/II binding pocket of KRAS G12V (Figure 1).^5^

Using a combination of saturation transfer difference (STD)^24^ and heteronuclear single quantum coherence (HSQC)^25^ NMR screenings, several fragments have been identified as binders in the switch I/II binding pocket. We measured the affinity of the best hits and solved the 3D KRAS-fragment structures using *N*MR^2^. In addition to showing how we solve complex structures using a conventional isotope-filtered NOESY pulse sequence,^26,27^ we present a NOESY pulse sequence with a relaxation filter, allowing *N*MR^2^ structure calculations with unlabeled material.

## Materials and Methods

### Protein Expression and Purification

KRAS G12V on a pet28a(+) vector was transformed with BL21* DE3 cells. After incubating overnight in LB, the cell pellet was diluted into a ^15^N or ^13^C,^15^N-labeled minimal medium. After reaching an OD of 0.8, the cells were induced with 0.5 mM IPTG, and the temperature was reduced to 18 °C. After 18 h, the cell pellet was harvested, resuspended in a buffer containing 50 mM Tris, 200 mM NaCl, 5 mM beta-mercaptoethanol (BME), 5 mM MgCl_2_, 10 mM imidazole, and protease inhibitor cocktail, pH = 7.4, and lysed with a microfluidizer. The lysate was centrifuged, filtered, and purified with a His-trap. KRAS was eluted with the same buffer containing 500 mM imidazole. After dialysis against imidazole-free buffer, the His-tag was cleaved overnight with TEV protease, and the reaction was purified by His-trap.^13^

### GMP-PNP Loading

KRAS was carefully diluted into a buffer containing 20 mM Tris, 0.1 mM ZnCl_2_, and 10 mM (NH_4_)_2_SO_4_. A 5 unit/mg amount of KRAS of liquid alkaline phosphatase and a 2× molar excess of GMP-PNP Li salt were added, and the reaction was gently shaken at 4 °C overnight. The buffer was exchanged to the NMR buffer containing 20 mM HEPES, 100 mM NaCl, 5 mM MgCl_2_, and 2 mM TCEP, pH 7.4. The loading was controlled by [^15^N,^1^H]-HSQC.

### Screening and Titrations

The STD-NMR and [^15^N,^1^H]-HSQC screening of the DSI-poised fragment library was performed on a Bruker Avance III HD 600 MHz spectrometer equipped with a cryoprobe and SampleJet. For the STD screening, the fragments were pooled in mixtures of six with a concentration of 600 μM per ligand, resulting in a total fragment concentration of 3.6 mM per sample. The KRAS G12V GMP-PNP concentration was 12 μM, giving a protein to ligand ratio of 1:50. The experiment was carried out in a deuterated NMR buffer containing 20 mM Tris-D_11_, 100 mM NaCl, 5 mM MgCl_2_, and 5 mM deuterated BME at a pD of 7.4. The STD-NMR experiment was done with an off-resonance pulse of 60 ppm, an on-resonance pulse of −1 ppm, and 256 scans with a 3 s and 100 Hz saturation pulse. Analysis was performed in TopSpin 4.1 and CcpNMR 3.1.^28^

The [^15^N,^1^H]-HSQC screening was done with a ^15^N-labeled KRAS concentration of 140 μM and a ligand concentration of 1 mM. The 2D spectra were measured with 184 (*t*_1_, max (^15^N) = 42 ms) × 2048 (*t*_2_, max (^1^H) = 121.7 ms) data points with 8 scans per increment and 0.8 s interscan delay. Processing was performed with a shifted cosine window function of both dimensions. Data analysis was performed in CcpNMR v 3.1.

All [^15^N,^1^H]-HSQC titration experiments were performed with ^15^N-labeled KRAS at either 500, 600, 700, or 900 MHz with ligand concentrations ranging from 5 to 5500 μM. A detailed summary for each ligand can be found in the Supporting Information. The NMR data were processed with nmrpipe, and the affinity was analyzed with TITAN.^29^

### NOESY Measurements, Structure Calculation, and Refinement

All filtered NOESY measurements were conducted on a 600, 700, or 900 MHz Bruker magnet. Either a ^13^C,^15^N-filtered [^1^H,^1^H]-NOESY or a T_1_,T_2_-filtered [^1^H,^1^H]-NOESY was acquired for each fragment. Typical fragment concentrations ranged from 3 to 6 mM, and the KRAS G12V concentration was typically 1 mM. All experiments were performed in the deuterated NMR buffer. Typical NOESY mixing times ranged from 20 to 120 ms. A detailed summary of all measurements performed can be found in the Supporting Information.

Peak picking was performed with Ccpnmr 3.1, and data analysis in R-Studio. Distances were calculated from the NOE build-up curves with the isolated two-spin system assumption. The cross-peak intensities *I_ij_*(*t*) were normalized with the diagonal peak *I_ii_*(*t*) according to Pokharna et al.^30^ The cross-relaxation rate σ_*ij*_ was then calculated with
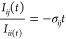
1with
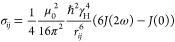
2and
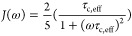
3as spectral spin density, μ_0_ as the permeability of the vacuum, *ℏ* as the reduced Planck constant, γ_H_ as the proton gyromagnetic ratio, and τ_c,eff_ as the effective correlation time of the protein–ligand complex.

*N*MR^2^ software was used for initial structure calculation and assignment, and CYANA^31^ was used for structure calculation. Structures were refined with the HADDOCK 2.4^32^ refinement options.

## Results and Discussion

### General Workflow

Figure 1 gives an overview of the adopted NMR pipeline to target oncogenic KRAS G12V, going from screening to SAR. Figure 1A shows the first step, in which the DSI-poised fragment library^33^ was screened against KRAS using STD-NMR experiments. To filter false-positive hits obtained by the STD-NMR screening and to collect information about the binding site, a secondary screen was conducted with [^15^N,^1^H]-HSQC chemical shift perturbation (CSP), shown in Figure 1B. The spectra overlap shows the apo state (blue) and the ligand-bound state (red), along with a close-up of residue G75. The HSQC screening identified not only the true positive hits and the location of the binding site but also provided insights into the potential ranking of the hits based on the magnitude of the chemical shifts, assuming similar binding modes for similar structural scaffolds. The affinities of the most promising hits were determined using ligand-titrated 2D-[^15^N,^1^H]-HSQC NMR, as shown in Figure 1C, and *K*_D_’s in the millimolar range were observed. To establish the structure–activity relationship (SAR), a protein-filtered NOESY was measured for the nine most promising compounds, as illustrated with the filtered NOESY example in Figure 1D. The cross-peaks obtained in the NOESY spectra were converted into distance restraints, a structure was calculated using *N*MR^2^, and the SI/SII (orange, red) binding site was confirmed. Based on the derived SAR, new compounds were selected following an SAR by the catalog approach, resulting in a moderate improvement in target engagement and affinities in the low millimolar range. This confirms that a 100% NMR-based drug discovery pipeline, including *N*MR^2^, is feasible and capable of coping with challenging targets.

### Screening and Affinity Determination

#### STD Screening

The DSI-poised fragment library comprises 890 molecules, designed such that analogues are readily accessible on the market and can be quickly synthesized. For the STD-NMR screening, the fragments were pooled into six mixtures, each at a concentration of 600 μM, resulting in a total fragment concentration of 3.6 mM with a protein:ligand ratio of 1:50. Hits were identified if a molecule exhibited a signal-to-noise (S/N) ratio greater than 5. From the 890 fragments screened, 133 hits were identified, corresponding to a 15% hit rate. A list of the SMILES for all hits is provided in SI Table 2. The STD spectra for fragment **5** are illustrated in Figure 1A (red), with the 1D spectrum of the mixture shown in blue. The STD spectra for the most promising hits are presented in SI Figure 1. Analysis of the core structures of the hits using DataWarrior^34^ revealed that most molecules possess a hydrophobic core.

#### HSQC Screening

To filter false-positive fragment hits and obtain information about the binding site, a secondary screening with chemical shift perturbation [^15^N,^1^H]-HSQC NMR was conducted using a 1 mM fragment and 110 μM KRAS. Approximately 30% of the hits were identified as false-positives or inconclusive due to significant pH changes upon addition, detected by the strongly shifting peak of Tris from the buffer in the 1D spectrum. The backbone assignment from the GTP-bound G12V mutant (BMRB entry 50773) was used to evaluate the chemical shifts. The complete [^15^N,^1^H]-HSQC spectra of KRAS without a binder (black) and in complex with fragment **5** (red) are shown in Figure 1C. A zoomed-in view of the peaks of the four predominantly shifting amino acids is presented in SI Figure 3. SI Figure 2A displays the CSP map of the backbone for the assigned residues of compound **1**, which exhibited the strongest shifts among all fragments. Unassigned residues are indicated by a 0 Hz shift. Shifts between 8 and 10 Hz are displayed in green, and those above 10 Hz are shown in blue. The switch I and switch II regions are colored orange and red, respectively. The HSQC peaks of the switch I and switch II regions are not observable potentially due to the high flexibility and conformational exchange of those amino acids.^35^SI Figure 2B shows the mapping of the shifts with their corresponding color code onto the crystal structure 6XHA. As illustrated in the CSP plot, the maximal shift observed is 50 Hz for T74. Only a few shifts are above 10 Hz. These small chemical shift changes at 1 mM ligand concentration indicate weak binding, presumably in the millimolar range. The strongest shifts are observed in residues L6, V7, L56, T74, and G75, which form a binding pocket between the switch I and switch II regions, a site that has been extensively studied in previous works.^10,13^ The shallow binding site is visible in the crystal structure (PDB 6XHA) shown in SI Figure 2B, where the blue surface between switches I and II represents the binding site with the strongest chemical shift changes. Additionally, some weaker shifts between 8 and 10 Hz are observed between residues 130–150. These shifts are only slightly above the average noise level, making it challenging to draw definitive conclusions. SI Figure 4 shows the chemical structures of the most promising hits identified in the secondary screening. Based on their structures, these hits are categorized into two classes: Class 1 comprises molecules with an indole-like 6–5 ring pattern (indole, purine, benzofuran, and benzothiazole), characterized by a hydrophobic pattern reported earlier. Class 2 molecules have a benzene ring as the hydrophobic moiety attached to an NH group, which presumably functions similarly to the NH group of the indole ring. The strongest shifts observed (SI Figure 3) are for class 1 fragments **1**, **2**, and **3**. Class 2 molecules, displayed in SI Figure 3 (fragments **4**, **5**, **7**, **10**, and **12**), show a particularly weak shift of V7 compared to class 1 molecules. The molecules presented in SI Figure 4 were selected for further investigation.

#### Affinity Determination

To further characterize the binding, a [^15^N,^1^H]-HSQC titration of the 13 fragments listed in Table 1 was performed. The amide signals of L6, V7, T74, and G75 were analyzed using the line shape analysis software TITAN.^29^ Additionally, the chemical shift perturbation binding curve was analyzed. An example of the binding curve and signal shifting for compound **1** is shown in Figure 1C. SI Figure 5 includes a zoomed-in view of residues L6, V7, T74, and G75 and the binding curves for T74 and G75 for all compounds from which a structure was later derived. The affinities were found to be in the low millimolar range for all compounds. However, no saturation was reached; therefore, the derived *K*_D_ values should be interpreted with caution. Therefore, the molecules should not be ranked solely based on their TITAN *K*_D_ values, but also on the curvature of the binding curve and, in the case of high structural similarity, on the magnitude of the chemical shift change, as a similar binding pose can be assumed. From the affinities derived with TITAN, listed in Table 1, it is evident that class 1 molecules tend to have higher affinities than class 2 molecules, which is supported by their stronger chemical shift changes. Bulky moieties such as methoxy (fragment **10**) and chloride groups (fragment **12**) attached to the hydrophobic ring system appear to lower the affinity and the chemical shift change. However, when small groups such as fluoride (fragments **2**, **3**, and **5**) are introduced, no significant change in *K*_D_ and CSP is observed compared to their counterpart (fragment **9**).

**Table 1:** Summary of All Compounds for which the Affinity and/or Structure with KRAS Was Determined

**fragment**	**SMILE**	**STD**	**CSP**	**class**	*K*_**D**_**[mM]**	**#NOE**	**PDB code**
**1**	C(c1ccco1)Nc1nc(cccc2)c2[nH]1	√	√	1	1.2	11	8QDK
**2**	CN(C)C(c1cc2cccc(F)c2o1)=O	√	√	1	1.6	8	8QDN
**3**	CC(NCCc1c[nH]c(cc2)c1cc2F)=O	√	√	1	1.8	10	8QDP
**4**	N*=*C1SC=CN1CC(Nc(cc1)ccc1F)=O	√	√	2	10	11	8QDS
**5**	Cc1n[nH]cc1CNc(cccc1)c1F	√	√	2	5.2	13	8QDT
**6**	*Cc*(cc1)cc(NC(Nn2cnnc2)=O)c1OC	√	√	2	15	0	
**7**	Nc(cc1)ccc1S(Nc1ccccc1)(=O)=O	√	√	2	5.9	0	
**8**	O=C(Nc1ccccc1)Nc1cnccc1	√	√	2	9	0	
**9**	CNCc1cn(-c2ccccc2)nc1.Cl	√	√	2	3.3	10	8QDW
**10**	COc(cc1)cc2c1sc(N)n2	√	√	1	5.9	0	
**11**	CS(Cc1nc(cccc2)c2[nH]1)(=O)=O	√	√	1		11	8QE7
**12**	O=C(CN1CCCC1)Nc1cccc(Cl)c1	√	√	2		13	8QEI
**13**	CN(C)Cc1c[nH]c(cc2)c1cc2OC	√	√	1		9	8QE6
**14**	CC=1C=C2C=CNC2=CC1C		√	1	3.2		
**15**	O=C1CNC(CN1)C2=CC=3C=CC=CC3N2		√	1	3.04	17	8PI0
**16**	OC(=O)C=CC1=CNC2=CC=CC=C12		√	1	4.5	8	8PIY
**17**	O=C1CCC(N1)C2=CC=3C=CC=CC3N2		√	1	4.6	12	8QEJ

#### *N*MR^2^

To establish a structure–activity relationship (SAR) for the most promising hits shown in Table 1 and SI Figure 4, a protein-filtered [^1^H, ^1^H]-NOESY spectrum was measured for the compounds listed in Table 1. The NOESY of fragments **1**, **4**, and **5** were measured with a [^13^C,^15^N]-filtered NOESY using ^13^C,^15^N-labeled KRAS, while fragments **2**, **3**, and **6**–**13** were measured with a T_1_,T_2_-filtered NOESY using unlabeled KRAS. Unlike the isotope filter, which filters out unwanted signals from the protein by labeling the protein and not the ligand, the T_1_ and the T_2_ filters rely on the different tumbling times of the ligand versus the protein. The protein has an approximately 100-fold slower tumbling time than the ligand and relaxes much faster, effectively removing unwanted protein signals.

**Figure 2:**
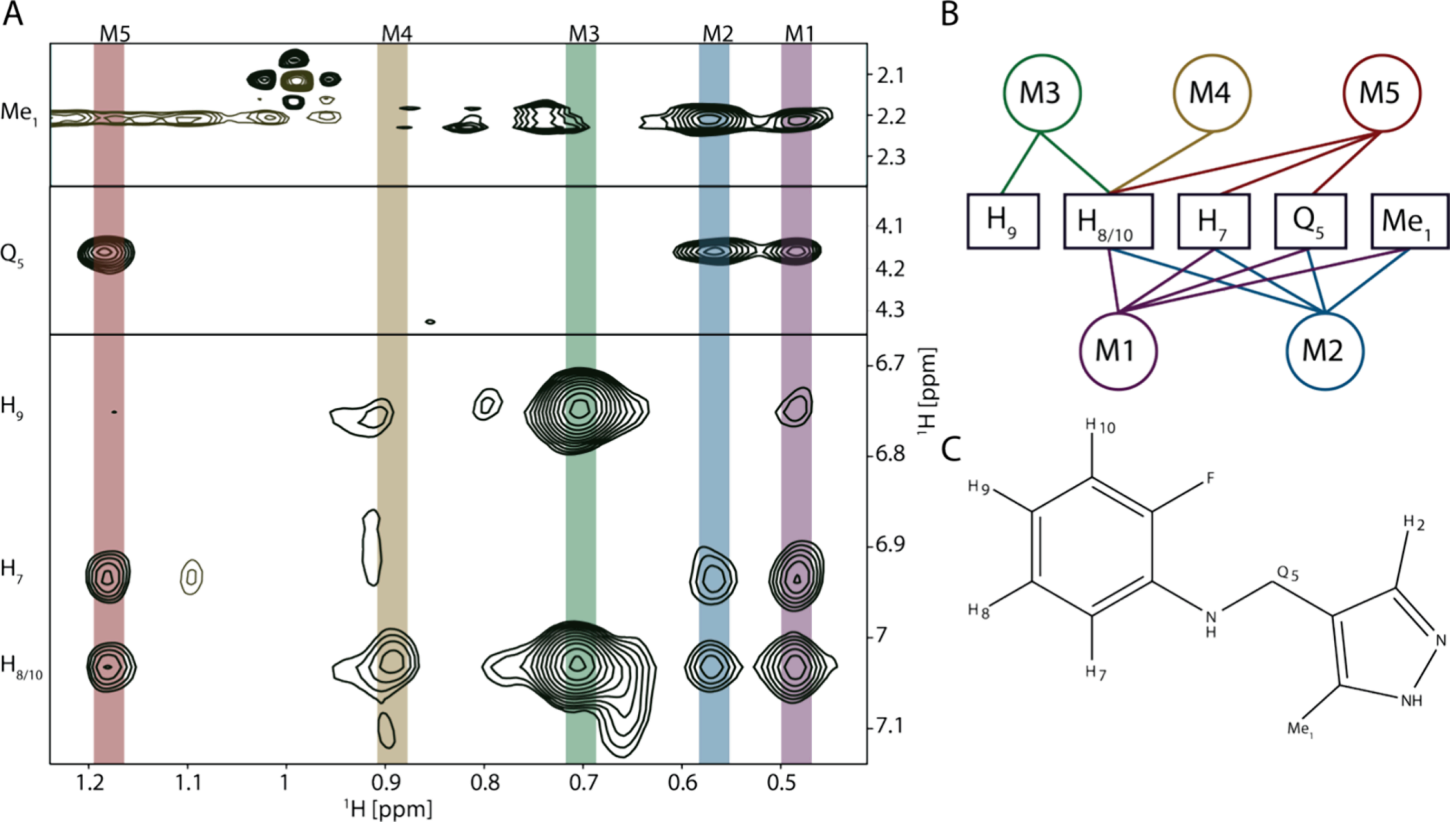
(A) Zoom into the intermolecular cross-peak region of the [^13^C,^15^N]-filtered [^1^H,^1^H]-NOESY spectrum of KRAS G12V GMP-PNP (^13^C,^15^N-labeled) and **5** (unlabeled) shown on the left. The zoomed-in view presents the aromatic region, the CH_2_ (Q_5_) peak, and the methyl peak Me_1_ of fragment **5**. The methyl groups of KRAS M1-M5 showing cross-peaks with respect to **5** are marked with different colors. (C) Distance network of the KRAS methyl’s M1–M5 and **5** and (B) chemical structure of **5** shown with the corresponding color code on the right.

Figure 2 illustrates the workflow used to derive a structure from the NOESY spectrum. Figure 2A shows a zoomed-in view of the methyl region of an isotope-filtered NOESY spectrum of KRAS and compound **5**. The methyl groups M1–M5 show cross-peaks to compound **5** and are marked with different colors. The NOESY spectra for all compounds that showed intermolecular cross-peaks are presented in SI Figures 6–14. Fragments **6**–**8** did not show any intermolecular cross-peaks.

Assuming a tumbling time (τ_c_) of 12 ns for KRAS, the effective tumbling time (τ_c,eff_) for a millimolar binder, calculated as a weighted average of the ligand and protein tumbling times, is significantly shorter, approximately 1 ns. As a result, the corresponding NOE signals are weak, approaching the detection limit, and are largely insensitive to changes in the affinity constant. Given the high uncertainty of affinity measurements due to the very weak binders, using the standard formalism to calibrate distance restraints becomes less reliable and relying on them for structure calculations would introduce substantial errors. To mitigate this, ligand–protein distance restraints were set to a fixed median distance of 4.5 Å.^36^ This approach consistently yielded effective tumbling times in the nanosecond range across all complexes.

Since the assignment of methyl groups M1–M5 was unknown, *N*MR^2^ software was employed to perform individual fragment structure calculations. Using distances extracted from the NOE buildups, a distance restraint map was constructed, detailed in Figure 2C and SI Figures 6–14. Given the relatively low number of distance restraints (9–13) compared to previously published *N*MR^2^ works, additional information was essential for successful *N*MR^2^ calculations.^37^ The binding site, identified through CSP mapping, revealed few methyl groups in the proximity. V7, L56, T58, M72, and T74 were identified as potential methyl groups capable of forming NOE cross-peaks with the ligand. The [^13^C,^1^H]-HSQC chemical shift perturbation (SI Figures 6–14) indicated no involvement of methionine in NOE cross-peaks. M1 exhibited no corresponding peak in the constant time (ct)-[^13^C,^1^H]-HSQC spectra (SI Figures 6A, 7A, 8A, and 10), but a weak peak was visible in conventional [^13^C,^1^H]-HSQC (SI Figure 15A). When compared to GDP-loaded KRAS, M1 exhibits a signal approximately 10 times stronger than that of the GTP-analogue KRAS complex (SI Figure 15B). This observation is consistent with the reported spin relaxation data, which indicate increased conformational exchange in the switch regions of the active form of KRAS,^35^ as well as the proximity of M1 to these regions. However, the line-broadening effects are more pronounced in the ^13^C dimension than in the ^1^H dimension, as evidenced by the cross-peaks in [^1^H,^1^H]-NOESY spectra. Based on chemical shifts, M1 and M2 were identified as leucine or valine. Assigning M3 and M4 was not possible, while M5 exhibited a characteristic chemical shift indicative of threonine, which is further supported by the strong [^15^N,^1^H]-HSQC chemical shift changes of T74. Subsequently, the *N*MR^2^ calculations were carried out with the partial assignment of M1 and M2 = Leu or Val and M5 = T74. All structure calculations were completed in less than 20 min, and M1, M2, M3, and M4 were assigned by the *N*MR^2^ algorithm to L56 QD2 and D1 and V7 QG2 and QG1, respectively. The same assignment was corroborated by 8 *N*MR^2^ structure calculations and propagated across all fragments for their respective structure determinations. Most fragments displayed NOE cross-peaks exclusively with the hydrophobic aromatic moiety of KRAS. Fragments, where the hydrophobic ring was connected to a second ring, exhibited flexibility or were constrained by intramolecular restraints. The refinement of these structures was executed using the web interface of HADDOCK v 2.4. The PDB codes for each fragment–protein structure that was calculated are summarized in Table 1.

To further assess the quality of the *N*MR^2^ methyl assignments and their subsequent structure calculation, a [^13^C,^15^N]-filtered NOESY of an analogue described by Sun et al.^10^ was measured in the presence of KRAS G12V in the GDP state. The molecular structure of the analogue, [^13^C,^1^H]-HSQC and NOESY spectra, along with the corresponding NOE build-up curves and distance restraint networks, are shown in SI Figure 16. In addition to the detection of strong cross-peaks at V7 and T74, NOEs from the benzimidazole ring to M67 were visible. The calculated structure matched the X-ray structure of 4EPY (SI Figure 15) and 4EPV (not shown), validating the *N*MR^2^ approach.

#### SAR

**Figure 3:**
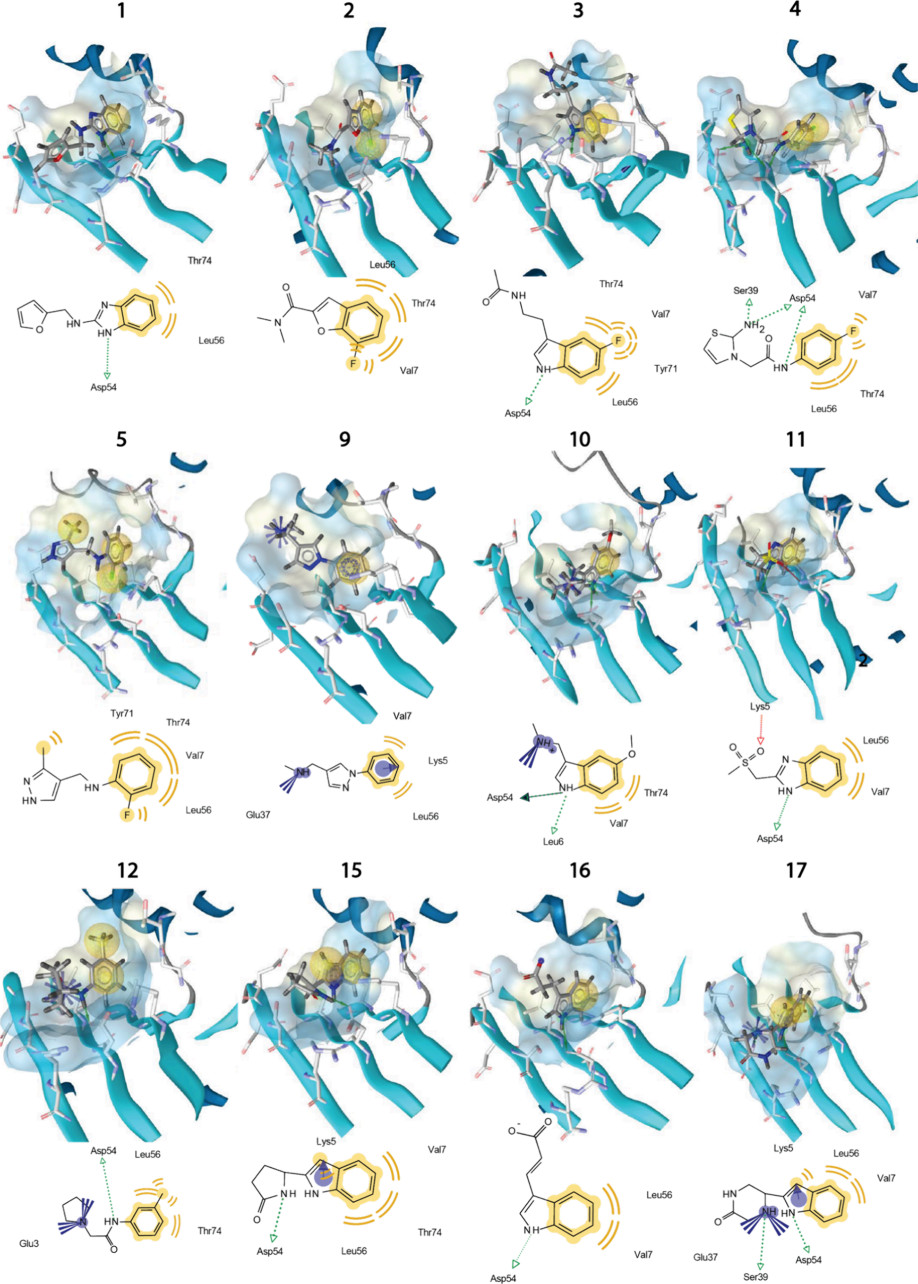
*N*MR^2^ structure calculation of KRAS G12V in the GMP-PNP bound form in complex with different fragments. The complex structures derived with *N*MR^2^ and CYANA in the binding pocket between the SI and SII region for fragments **1**–**5**, **9**–**12**, and **15**–**17** are presented after MMFF94 energy minimization in LigandScout. The corresponding molecular interactions are presented in 2D and 3D views.

LigandScout software was utilized to generate pharmacophores based on the structures derived from the fragments.^38^ MMFF94 energy minimization of both ligand and protein structures was performed within LigandScout. The generated pharmacophores for each fragment–protein structure are visualized in 2D and 3D formats in Figure 3. As anticipated from the CSP analysis, the hydrophobic pocket formed by K5, V7, L56, Y71, and T74 is occupied by the aromatic moiety in both class 1 and class 2 molecules. Specifically, the indole proton of class 1 molecules or the NH proton of class 2 molecules forms a hydrogen bond with D54. Interactions involving other parts of the molecule should be interpreted cautiously due to the limited distance restraints in those regions. Based on their affinities, class 1 molecules exhibit a preference over class 2 molecules. Despite both being capable of forming a hydrogen bond with D54, as depicted in Figure 3, class 1 molecules are constrained in their conformations, which are entropically favorable. Additionally, bulky moieties on the aromatic ring, such as the methoxy group in fragment **10** or the chloride group in fragment **12**, distort their orientation within the pocket. In summary, the key interactions with KRAS identified in the pharmacophores generated from the nine *N*MR^2^ structures include a hydrophobic moiety and the potential to form a hydrogen bond with D54. These findings suggest that it is feasible to design new molecules and characterize their pharmacophores using *N*MR^2^.

#### New Molecules

To identify fragments with improved affinities compared to those found in the initial screening, new fragments were designed based on the predominant findings of the pharmacophore. These new fragments maintain the indole moiety to ensure the hydrophobic interaction and hydrogen bond with D54. Their structures are presented in SI Figure 17. Fragment **14** features an extended hydrophobic moiety with two symmetric methyl groups, potentially enhancing hydrophobic interactions without altering its orientation in the binding pocket-like molecules **10** and **12**. Fragments **15** and **17** both have additional protons bonded to nitrogen at position 2 of the indole ring, which could potentially form additional hydrogen bonds with D54. Furthermore, the carbonyl groups in these fragments could interact via hydrogen bonding with S39. Fragment **16** extends from position 3 of the indole with a carboxyl group that might form a hydrogen bond with T74, which is in the proximity. The affinities of these new fragments were determined using ligand-titrated [^15^N,^1^H]-HSQC CSP. The chemical shift changes of L6, V7, T74, and G75 for fragments **15**, **16**, and **17** are shown in SI Figure 19, along with the binding curve analysis for T74 and G75. To compare their chemical shift maps with KRAS in complex with 1 mM of fragment **1** (SI Figure 2), the titration point of 800 μM of fragment **15** was used to generate a chemical shift map, shown in SI Figure 18. The magnitude of the chemical shift changes for fragment **1**; and fragment **15** is comparable. Notably, fragment **15** starts to saturate, suggesting that the affinity of 3.1 mM derived with TITAN is more reliable than the affinity of 1.2 mM of fragment **1**. Moreover, by comparison of the chemical shift changes in the [^13^C,^1^H]-HSQC spectrum presented in SI Figure 20, T74 changes by 39 Hz upon the addition of fragment **15**, whereas T74 changes by 26 Hz upon the addition of fragment **1**. The structures of fragments **15**, **16**, and **17** bound to KRAS GMP-PNP were calculated as described in Figure 2. The NOESY spectra of fragments **15** and **16** were measured with [^13^C,^15^N]-filtered NOESY, while the NOESY spectrum of fragment **17** was measured with a relaxation filter. The spectra of these fragments, their NOE buildups, and corresponding distance restraint maps are shown in SI Figures 20–22. The 2D and 3D pharmacophores generated by LigandScout are shown in the last row of Figure 3. As expected, the aromatic moiety forms interactions with amino acids K5, V7, L56, and T74, and the hydrogen bond of the indole to D54 is prominently featured again. With restraints applied to the residues of fragments **15** and **16**, they exhibit less flexibility in structure calculations, increasing confidence in their pharmacophore representations in Figure 3.

No hydrogen bond was observed with the carbonyl group, as expected earlier. However, the amine group in the 6-ring forms a hydrogen bond with D54. Based on the affinity data, binding curves, and chemical shift perturbation analysis, newly designed structures **15** and **17** exhibit similar or slightly better binding properties compared to the best hits **1**–**3** identified in the initial screening. This highlights the capability of *N*MR^2^ to aid in early-stage lead design, where X-ray crystallography may not provide sufficient insights.

#### Structure Calculation Using Unlabeled Protein

**Figure 4:**
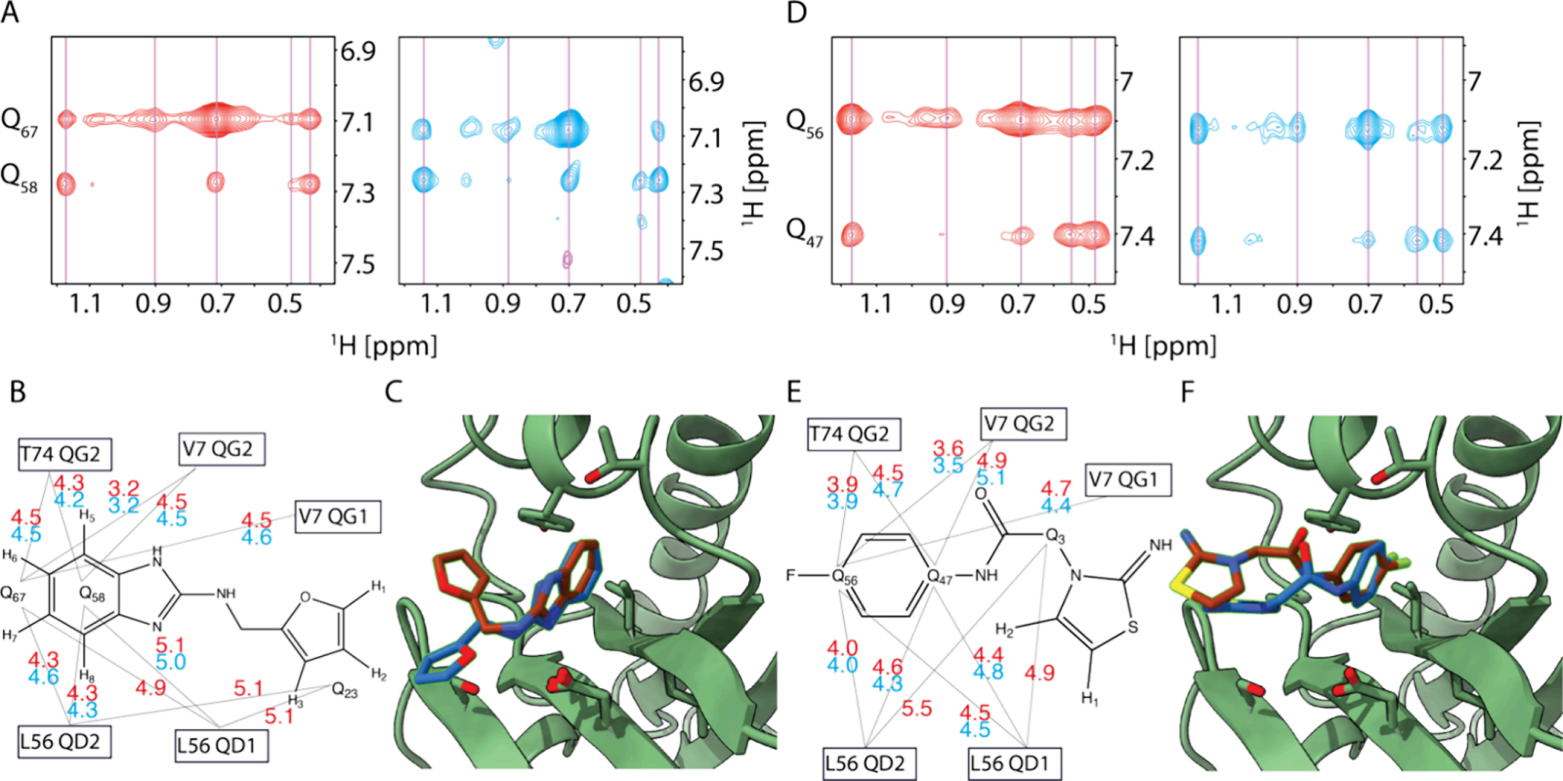
*N*MR^2^ complex structure calculation based on isotope- or relaxation-filtered NOESY for KRAS G12V GMP-PNP. [^13^C,^15^N]-filtered [^1^H,^1^H]-NOESY (red) and T_1_,T_2_-filtered [^1^H,^1^H]-NOESY (blue) spectrum presented for KRAS in complex with (A) **1** and (D) **4**. The corresponding distance network with annotated distances in Å is shown in (B) and (E), and the overlap of the calculated structure for the two filters is presented in (C) and (F).

The complex structures of fragments **1**, **4**, **5**, **15**, and **16** depicted in Figure 3 were derived using data obtained from [^13^C,^15^N]-filtered NOESY experiments employing ^13^C and ^15^N-labeled proteins. For the remaining structures, novel pulse sequence utilizing relaxation-filtered NOESY was employed, which does not require protein labeling. This innovative pulse sequence takes advantage of the faster relaxation properties of proteins compared to small molecules typically found in fragment-based drug discovery. This is particularly beneficial as initial discoveries in FBDD often involve weak binders where the ligand retains relaxation properties similar to its free form. In this relaxation-filtered NOESY approach, an inversion recovery pulse block serves as a T_1_ filter, followed by a perfect echo sequence and a CPMG without J-modulation, as a T_2_ filter.^39^ The pulse sequence is shown in SI Figure 23. These filters effectively remove most of the protein signal, with any residual protein signal discernible by comparing the NOESY spectra of different ligands. Figure 4A,D illustrates a zoomed-in view into the NOESY spectra of ligands **1**–**4** in the presence of KRAS: The red spectra depict results from isotope-filtered NOESY using double-labeled KRAS, while the blue spectra show data from relaxation-filtered NOESY with unlabeled protein. Despite the different pulse sequences and sample conditions, both ligands exhibit nearly identical NOE spectra. Figure 4B,E presents interaction maps between the methyl groups of KRAS and ligands **1** and **4**. Distances obtained from isotope-filtered or relaxation-filtered NOESY are colored red and blue. Minor differences are observed; the isotope-labeled NOESY shows slightly higher sensitivity, as evidenced by the detection of interactions with more distant residues like Q_23_ for ligand **1** and Q_3_ for ligand **4**. However, such distance restraints can also be measured in relaxation-filtered NOESY with longer mixing times. Finally, structures calculated based on these interaction maps for ligands **1** and **4** are depicted in Figure 4C,F, with red representing isotope-filtered NOESY and blue representing relaxation-filtered NOESY. The rigid core ring structures overlap closely for both fragments, demonstrating consistency between the two methods. The second ring moiety, where less information is available, exhibits flexibility and thus varies between the two structures. Overall, the overlap demonstrates that relaxation-filtered NOESY pulse sequences offer a viable means of computing complex structures without the need for protein labeling. This approach is particularly advantageous in scenarios where isotope labeling is challenging, such as in systems with low expression yields or expressed in mammalian cells.

## Conclusions

This work demonstrates the comprehensive use of NMR in a drug discovery pipeline encompassing screening, structure–activity relationship analysis, and hit optimization. While the screening methods employed are well-established in fragment-based drug design, this study marks the first application of *N*MR^2^ in such a drug discovery workflow.

*N*MR^2^ successfully determined the complex structures of the most promising hits from two distinct hit classes identified in this study, both located within the extensively studied binding site between the switch I and II regions of KRAS G12V. The pharmacophore model derived from these *N*MR^2^ structures facilitated the design of new ligands through a structure–activity relationship by a catalog approach, achieving similar or slightly improved affinity.

Moreover, this study introduces a novel pulse sequence, relaxation-based filtered NOESY, which enables *N*MR^2^ structure calculations without the necessity of protein labeling. This advancement broadens the scope for medicinal chemists to investigate systems previously inaccessible due to challenges in crystallization or unsuccessful X-ray structure determination. Importantly, it eliminates the requirement for isotope-labeled samples in generating NMR structures, thereby enhancing the applicability of NMR in studying a wider range of biological systems.

## Supporting Information Available

Extended materials and methods with all listed NMR experiments and relevant settings; (SI Table 2) list of the SMILES from all hits found in the STD-NMR screening; (SI Figure 1) STD-NMR spectra for all compounds; (SI Figure 2) chemical shift perturbation of KRAS binding to **1**; (SI Figure 3) CSP of the most promising hits in the secondary screening; (SI Figure 4) classification of the most promising hits in either class 1 or class 2 based on their structure; (SI Figure 5) binding affinity curves for some hits; (SI Figures 6–14) information used for structure calculation, including protein-filtered 2D NOESY spectra, [^13^C,^1^H]-HSQC spectra, and distance restraint networks; (SI Figure 15) [^13^C,^1^H]-HSQC CSP comparison of M1 and M2 in the GDP and GMP-PNP state; (SI Figure 16) all relevant information used to calculate the *N*MR^2^ structure of the positive control; (SI Figure 17) compound generated by SAR by catalog; (SI Figure 18) CSP mapping; (SI Figure 19) affinity binding curves; (SI Figures 20–22) all relevant information used for the structure calculation of those new molecules; (SI Figure 23) T_1_,T_2_-filtered NOESY pulse sequence (PDF)
