# Supplementary material for: NMR2-Based Drug Discovery Pipeline Presented on the Oncogenic Protein KRAS
Source: J Am Chem Soc. 2025 Apr 14;147(16):13200–9. doi: 10.1021/jacs.4c16762 (PMC12022975; doi:10.1021/jacs.4c16762)
Supplement: Supplementary file 1 — ja4c16762_si_001.pdf [file ja4c16762_si_001.pdf]

SI Information: An *N*MR²-based drug discovery pipeline presented on the oncogenic protein KRAS

Matthias Bütikofer[a,b], Felix Torres [b,c], Harindranath Kadavath[b,d], Nina Gämperli[b], Marie Jose Abi Saad[a], Daniel Zindel[b], Nicolas Coudevylle[a], Roland Riek*[b] and Julien Orts*[a]

[a] Department of Pharmaceutical Sciences, University of Vienna
Josef-Holaubek-Platz 2, 2F 353, A-1090 Vienna, Austria
E-mail: julien.orts@univie.ac.at

[b] Institute for Molecular Physical Science
Vladimir Prelog Weg 2, 8093, Zürich, Switzerland
E-mail: riekr@ethz.ch

[c] Dr. F. Torres
NexMR AG
Wiesenstrasse 10A, 8952, Schlieren, Switzerland

[d] St. Jude Children's Research Hospital
262 Danny Thomas Place
Memphis, Tennessee 38105, USA

*Correspondence to:
julien.orts@univie.ac.at

MATERIAL AND METHODS

TITRATIONS

1, 2, 3: The [^{15}N, ^{1}H]-HSQC experiments for determining affinities were measured with 232 ($t_{1,max}$ (^{15}N) = 53.0 ms) X 2048 ($t_{2,max}$ (^{1}H) = 121.7 ms) data points with 20 scans per increment and 0.8 s interscan delay on a Bruker Avance IIII HD 600 MHz spectrometer equipped with a cryoprobe and SampleJet. The ^{15}N KRAS G12V concentration was 120 μM. The fragments were titrated at a concentration of 50, 75, 100, 200, 300, 400, 500, 1000, 1500 μM.

4, 5, 9: [^{15}N, ^{1}H]-sofastHSQC experiments were done on a Bruker Ascend console NEO 500 MHz with a prodigy probe head and a sample changer and 200 ($t_{1,max}$ (^{15}N) = 56.3 ms) X 1024 ($t_{2,max}$ (^{1}H) = 73.1 ms) data points were acquired. 0, 59, 100, 200, 400, 600, 800, 1093, 1180, 1289, 1483, 1575 and 1870 μM of **4** were titrated to 120 μM ^{15}N labelled KRAS G12V and 64 scans per increment were measured. 0, 50, 100, 200, 400, 700, 1155, 1600, 2050, 2500, 2900, 3250, and 3350 μM of **5** were titrated to 120 μM KRAS and 16 scans per increment were acquired. 0, 50, 100, 200, 400, 800, 1185, 1574, 2314 and 2861 μM of **9** were titrated to 120 μM KRAS with 4 scans per increment.

15, 16: The [^{15}N, ^{1}H]-HSQC experiments for determining affinities were measured with 240 ($t_{1,max}$ (^{15}N) = 34.7 ms) X 2048 ($t_{2,max}$ (^{1}H) = 71.2 ms) data points with 2 scans per increment and 0.9 s interscan delay on a Bruker Avance III HD 900 MHz spectrometer equipped with a cryoprobe. 0, 10, 30, 70, 115, 200, 410, 790, 1910, 3700, and 5500 μM of the fragment was titrated to ~100 μM KRAS.

17: The [^{15}N, ^{1}H]-HSQC experiments for determining affinities were measured with 256 ($t_{1,max}$ (^{15}N) = 52.5 ms) X 2048 ($t_{2,max}$ (^{1}H) = 121.6 ms) data points with 8 scans per increment and 0.9 s interscan delay on a Bruker Avance IIII HD 600 MHz spectrometer equipped with a cryoprobe.

NOESY MEASUREMENTS

Table SI1: Summary of all [^{1}H, ^{1}H] - NOESY spectra measured for all compounds which a NMR2 structure was derived.

COMPOUND	$T_{1,MAX}$ (^{1}H) [MS]	$T_{2,MAX}$ (^{1}H) [MS]	FILTER [MS]	MIXING TIME [MS]	SCANS	FIELD [MHZ]	LABELLING	~MEASUREMENT TIME PER NOESY [H]
**1 -LABELLED**	240	35.5	Isotope	50, 70, 90	160	600	^{13}C, ^{15}N	48
**1 - UNLABELLED**	436	20.4	T_1: 340<br>T_2: 124.5	60, 80, 100	88	700	None	30
**4 - LABELLED**	227	37.9	Isotope	70, 90, 110	160	600	^{13}C, ^{15}N	53
**4 - UNLABELLED**	95	20.8	T_1: 5ms<br>T_2: 306	80	112	900	^{13}C, ^{15}N	34
**5**	240	33.2	Isotope	70, 90, 110	160	600	^{13}C, ^{15}N	47
**15**	106	25.5	Isotope	5, 10, 20, 30, 60, 80, 100	160	700	^{13}C, ^{15}N	38
**16**	106		Isotope	5, 70, 90, 110	160	600	^{13}C, ^{15}N	43
**3**	478	24.3	T_1: 273<br>T_2: 184.4	60, 80, 90	80	600	None	24
**2**	426	19.8	T_1: 5<br>T_2: 305	60, 80, 100	96	900	None	28
**13**	106	22.9	T_1: 290<br>T_2: 127.7	20, 30, 90, 110	160	700	None	41
**9**	106	22.9	T_1: 290<br>T_2: 127.7	20, 90, 110	152	700	None	39
**11**	95	20.8	T_1: 5<br>T_2: 162	20, 30, 80, 110	160	900	None	45
**12**	95	20.8	T_1: 5<br>T_2: 162	20, 30, 80, 110	160	900	None	45
**17**			T_1: 5<br>T_2: 250	80, 100		700	None	44

RESULTS AND DISCUSSION

Table SI2 A list of smiles for all hits found in the STD screening showing a S/N ratio higher than 5.

Smile STD hit	Smile STD hit	Smile STD hit	Smile STD hit
**C(c1ccco1)nc1nc(cccc2)c2[nh]1**	Cn(cc1cc(f)ccc1)s(n)(=o)=o	Cc(c)(c1)occn1c(cc(f)(f)f)=o	N#cc(cc1)cc(f)c1n1ccncc1
**Cn(ccc1)c2c1ccc(s(n)(=o)=o)c2**	Cnc(c(cc1)ccc1s(n)(=o)=o)=o	Coc(cccc1)c1n1ccn(cc#n)cc1	Cs(n1[c@@h](cn)ccc1)(=o)=o
**Cn(c)c(c1cc2cccc(f)c2o1)=o**	Cs(n1ccn(cc2ccco2)cc1)(=o)=o	Cc1nc(cn(c=cc(c)=c2)c2=o)cs1	Cn(cc1c(o)=o)nc1-c1ccncc1
**Cc(nccc1c[nh]c(cc2)c1cc2f)=o**	Cnc(cc1)ccn1c(c1(ccc1)c(cc1)ccc1f)=o.oc(c(f)(f)f)=o	Cc(nc(ccc(oc(f)f)c1)c1f)=o	Ccnc(c(cc1)ccc1ns(c)(=o)=o)=o
**N=c1sc=cn1cc(nc(cc1)ccc1f)=o**	Cc([nh]nc1c)c1s(n(c)c)(=o)=o	Cc1nc(co)nc(n2ccccc2)c1	Cn(c)cc1ccc(c(o)=o)o1.cl
**Cc(cc1)cc(nc(nn2cnnc2)=o)c1oc**	Cc(c)(c)c(cc(cc1)o)c1o	Cn(c1cccccc1)s(c)(=o)=o	Cc(n(c)nc1c)c1ns(c1cc(f)ccc1)(=o)=o
**Cncc1cn(-c2ccccc2)nc1.cl**	Cn(c)c(n(cc1)ccn1c(n(c)c)=o)=o	Cc1c(cnc2nnnn2c)cccc1	C(c1nocc1)n1ccncc1
**Cc1n[nh]cc1cnc(cccc1)c1f**	Cc(c1cc1)n(c)c(c1c(c)ncs1)=o	Cn(c)c(c(ccc(f)c1)c1br)=o	Cc(nc1cccc2nonc12)=o
**Nc(cc1)ccc1s(nc1ccccc1)(=o)=o**	Occ1cn(-c(cc2)cc(cl)c2cl)nn1	Cc(ccc(-n1nnnc1)c1)c1n	Cc(n(c1)cc1c(o)=o)=o
**O=c(nc1ccccc1)nc1cnccc1**	Coc(n(cc1)ccn1c(cc1)ccc1f)=o	Cnc(cccc1)c1s(c)(=o)=o	Cs(nc1(ccccc1)c(o)=o)(=o)=o
**Cc(c)(co)nc(nc(cc1)ccc1cl)=o**	Coc(nc1nnc(cc2cccc2)s1)=o	Cc(c)n(c)s(n(ccn1)cc1=o)(=o)=o	Cc(c1nc(cc2ccccc2)no1)n.cl
**Cc1noc(c)c1cn1n=c(c)sc1=o**	O=c(c1nccnc1)nc1cccc(f)c1	Cc1(c)n(ccn)ccc1	C(c1cscn1)n1ccncc1
**Cc([nh]nc1c)c1-c(cccc1)c1n**	Cc(c)(cn(c1)c(nccc2)c2f)c1o	O=c(c(cc1)ccc1cl)n1ccscc1	Cc1nc(cn(c2)[c@h](cn)c[c@@h]2f)cs1
**Cnc(nc1nc(ccc(oc)c2)c2s1)=o**	Cc(c)c1nnc(ncc(c)(c)c)s1	O=c(c(n1ccccc1)=cn1)nc1=o	Cc(c(cc1)ccc1occ(o)=o)=o
**Nc(nc(cc1)ccc1oc(f)(f)f)=s**	Coc1ccc(c(c2ccccc2)n)cc1.cl	C(c1nc(c2cc2)no1)n1ccocc1	O=s(c1cc(f)ccc1)(nc(cc1)ccn1c1cc1)=o
**Coc(cc1)cc2c1sc(n)n2**	O=c(c(n1ccocc1)=cn1)nc1=o	Cc(ncc(cc(cc1)br)c1oc)=o	Cc(nc(c(oc)c1)cc(oc)c1cl)=o
**O=c(c1ccco1)nccc(cc1)ccc1f**	Cc(nc1nc(sc)ns1)=o	O=s1(ccn(cc2cccco2)cc1)=o	Occ1cn(-c(cc2)ccc2cl)nn1
**Cc(cs(n)(=o)=o)c1ccccc1**	Cn(c)c(cn1ncc2c1cccc2)=o	O=c(cn1ccncc1)nc(cc1)ccc1f	Cn(cco)c(c(f)c1)ncc1cl
**Cc(c1cn(-c2ccccc2)nn1)n.cl**	Coc(cc1)ccc1nc(nc1ccncc1)=o	Cc(c1nc(-c2c(c)onc2)cs1)oc	Ccc(c)(c)nc(c1cc(c)n[nh]1)=o
**Cc1ccc(cc(nc2nccs2)=o)cc1**	C[c@h](c1)o[c@@h](c)cn1c(coc(f)f)=o	Cc(c(cc1)ccc1nc(cn1cccc1)=o)=o	O=c(cc(cc1)ccc1cl)nc1ncccn1
**Cc(c(nc([c@h]1occc1)=o)ccc1)c1cl**	Coc(c(cc1)cc(n)c1s(n)(=o)=o)=o	O=c(cn1cccc1)nc(cc1)ccc1f	Cc(c)(c)nc(cn1nc(c(f)(f)f)cc1)=o
**Cs(cc1nc(cccc2)c2[nh]1)(=o)=o**	Cc(nc(cc1)ccc1n)=o	Cc1noc(cc(c2ccccc2)n)n1.cl	Ccc1noc(c)c1c(nc1noc(c)c1)=o
**O=c(cn1cccc1)nc1cccc(cl)c1**	Cn1nnc(nc(c(cc2)ccc2cl)=o)n1	Cc(c1cccc(nc(c(f)(f)f)=o)c1)=o	Cc1csc(cnc(c2ccc2)=o)n1

**Cc(n(cc1)ccn1c(cc1)ccc1cl)=o**	Cc(cc1)ccn1c(ccc(n)c1)c1cl.cl	Cc(c(o)=o)oc(cccc1)c1f	Cc(cc1)ccc1nc1ncnc2c1cn[nh]2
**Cc1noc(cn2n=c(c)c=cc2=o)c1**	Cc(n(c)nc1)c1c(ncc1ccc1)=o	Cc1nsc(n2ccnccc2)n1	Coc(cc1)ccc1nc(cn)=o.cl
**O=c(c1cc1)n(cc1)ccn1c(cc1)ccc1f**	Ccs(ncc(cc1)cc2c1oco2)(=o)=o	Cc1c(cn2ccncc2)c(c)nn1c	Cc(c)nc(c1cc(c)nn1c)=o
**Cc(c(cc1)ccc1occ(o)=o)=o**	Cc(c(cc1)ccc1-c1cncnc1)n	Ccn1nccc1cnc.cl.cl	Cc1ccc(cn(cc2)ccs2(=o)=o)cc1
**Cc(c(cc1)ccc1-c1cncnc1)n**	Fc(c1nnc(n2cccc2)s1)f	Cn(cc1)ccn1s(n1ccncc1)(=o)=o	Cs(nccc(cc1)ccc1f)(=o)=o
**Ncc(n(cc1)ccn1c(cc1)ccc1cl)=o**	Cc1cc(c(nc2cn(c)nc2)=o)no1	Cn(c)s(n1ccn(c)cc1)(=o)=o	Cc(c(c)c1)cc2c1ncn2cc(n)=o
**Cc1c(cs(n)(=o)=o)ccc(f)c1**	Ccn(c)c(nc1cnns1)=o	O=s(cc1)(ccc1n1ccncc1)=o.cl.cl	Cc1cc(n(c)c(c2)cc2o)nc(c)n1
**O=c(c=c1)nc=c1oc(f)f**	Cc(c(n)=o)n(cc1)ccn1s(c)(=o)=o	Coc1cc(cn)cc(oc)c1oc	Cc(ncc(cc1)ccc1s(n)(=o)=o)=o
**Cc(c1nc(cc2ccccc2)no1)n.cl**	Cc(nc1cc(c)n[nh]1)=o	Cc(nc(cc1)cc(n2)c1nc2=o)=o	
**Coc(n(cc1)ccn1c(cc1)ccc1f)=o**	Cc1noc(cn2n=c(c)c=cc2=o)c1	Cc(n(c1)cc2c1ccc(n)c2)=o	
**Cc1nc(cn(c2)[c@h](cn)c[c@@h]2f)cs1**	Coc(c1cccc(ns(c)(=o)=o)c1)=o	Cc(nc1c(cc(o)=o)cccc1)=o	

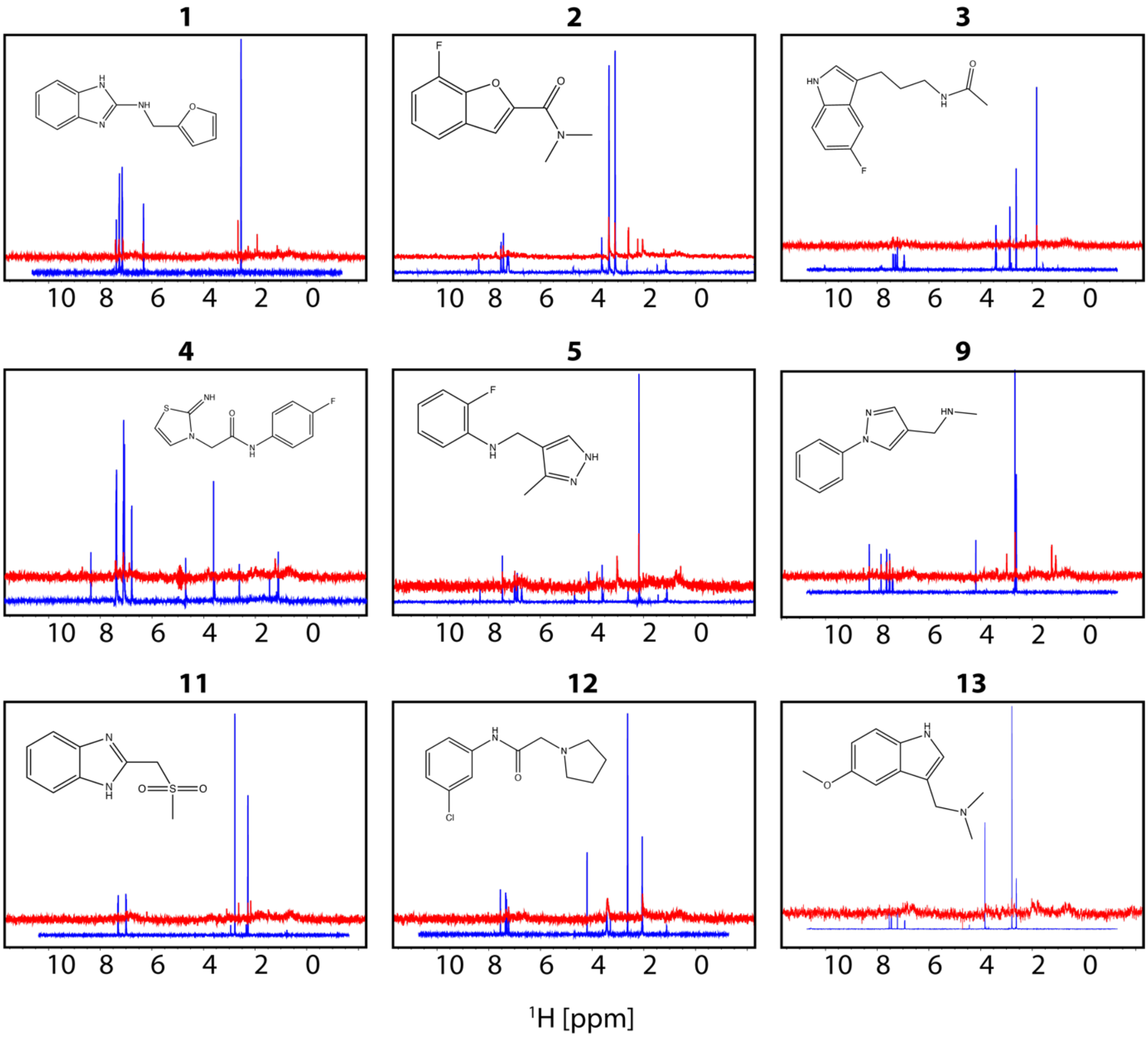

SI Figure 1. The STD-NMR spectra (red) and the 1D-NMR reference spectra (blue) of the primary screening are presented for compounds which a complex structure was derived.

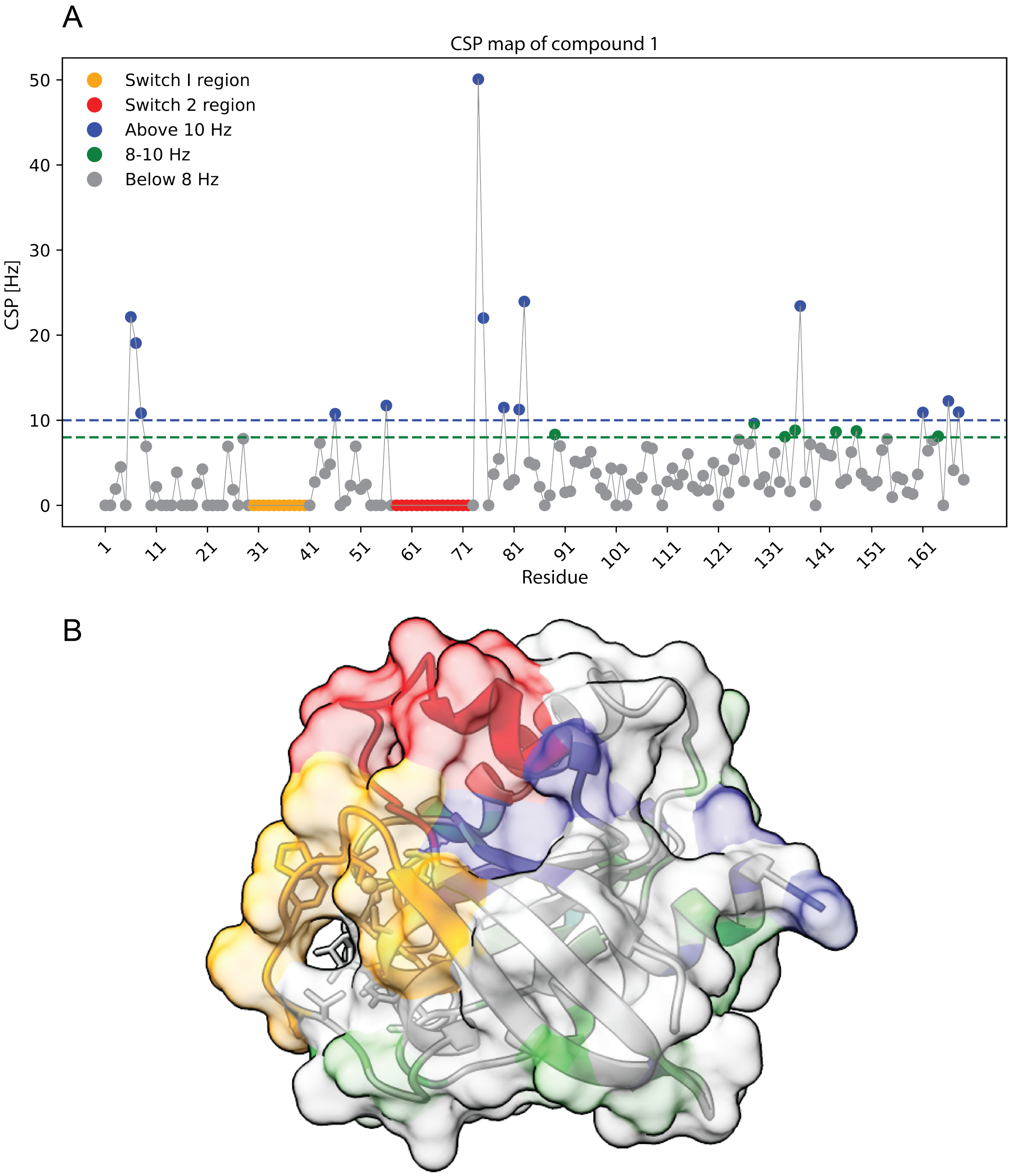

SI Figure 2. (A) Chemical shift perturbation map of KRAS G12V GMP-PNP upon addition of 1 mM of compound **1**. Shifts higher than 10 Hz are shown in blue and higher than 8 in green. Switch I and II regions are shown in orange and red, respectively. (B): Color coding mapped on crystal structure 6XHA.

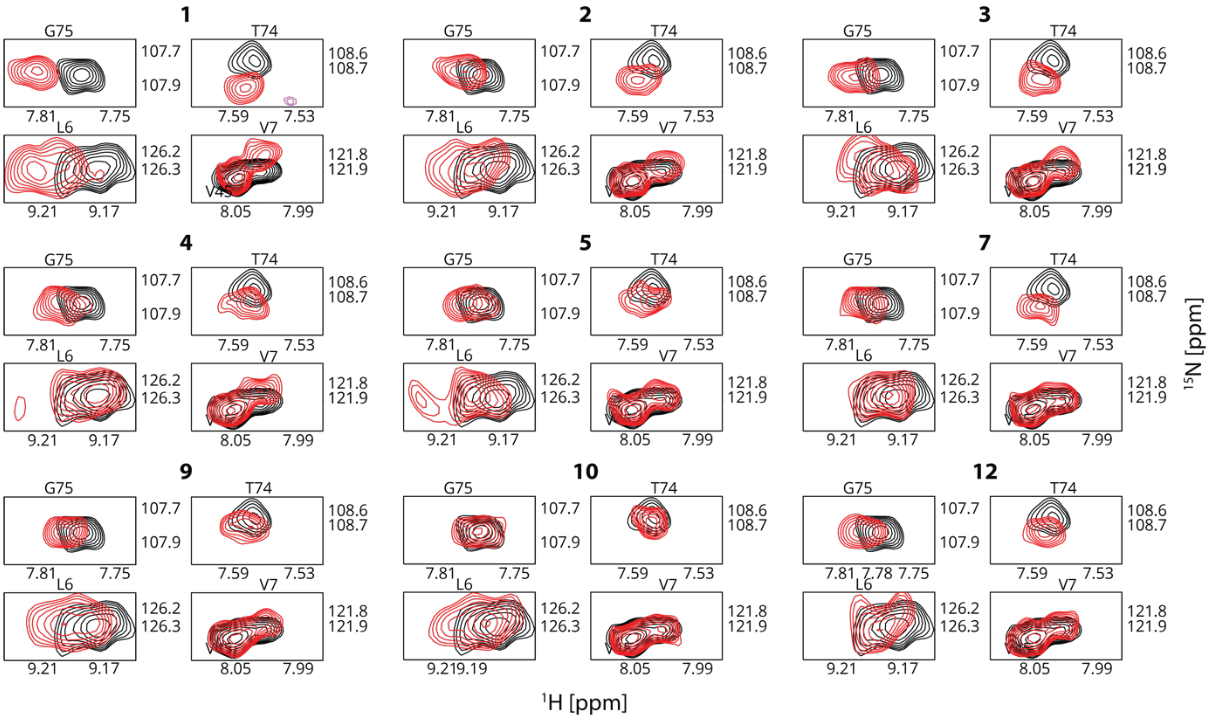

SI Figure 3. [^{15}N, ^{1}H]-HSQC chemical shift perturbation at a concentration of 100 µM KRAS G12V GMP-PNP in absence (black) and presence (red) of 1 mM fragment. A zoom into the regions of L6, V7, T74, and G75, located in the binding site, is presented. The compounds shown are among the fragments that showed the most promising shifts.

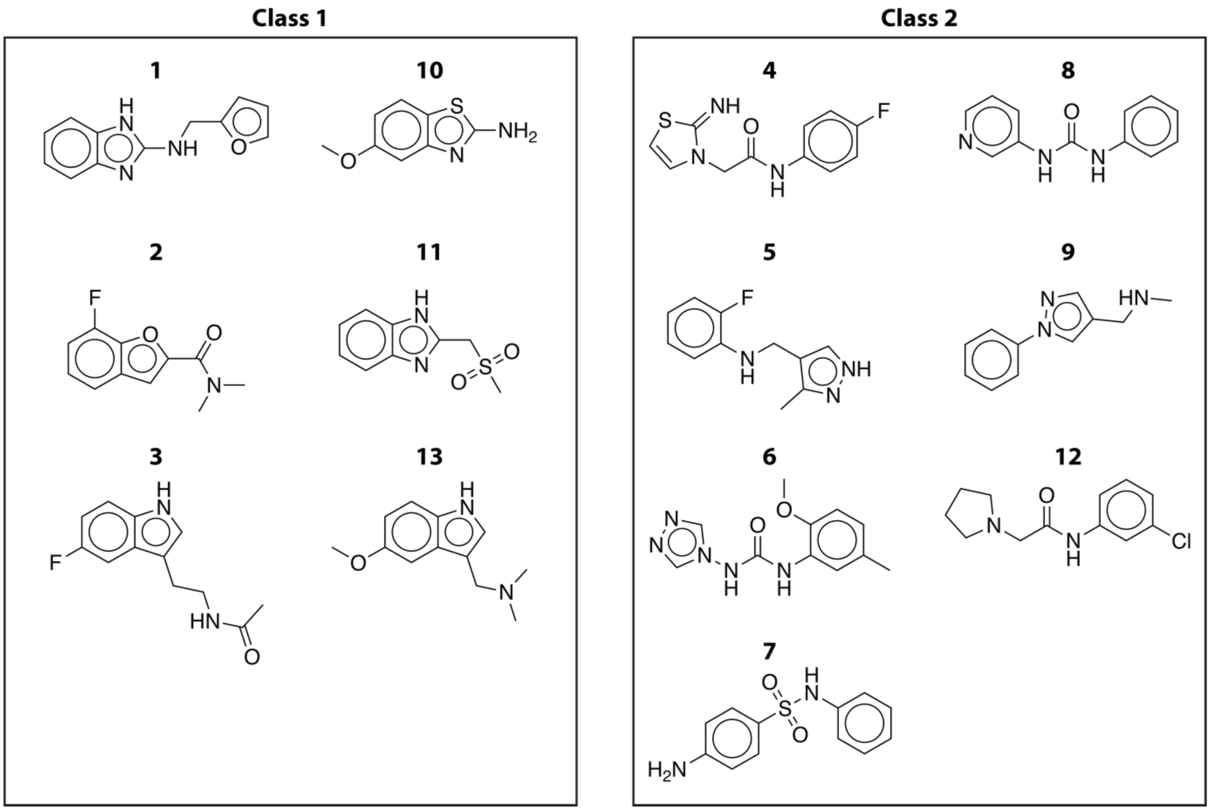

SI Figure 4. Chemical structure of the fragments **1**-**13**. The fragments showed the strongest chemical shift changes in the secondary HSQC screening. Class 1 molecules have an indole-like shape, and Class 2 molecules have an aromatic moiety connected to an NH group.

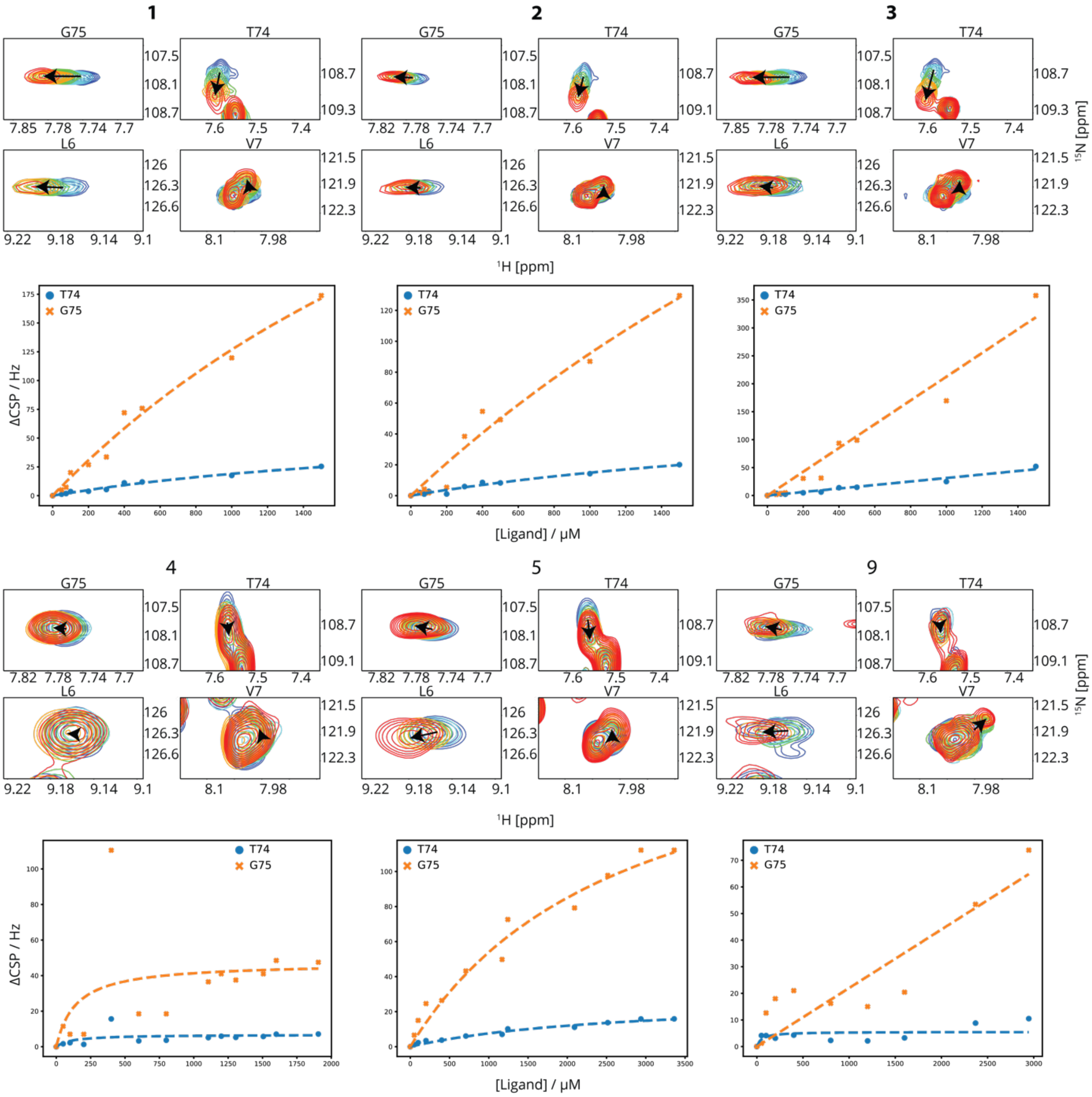

SI Figure 5. Ligand titrated [^{15}N, ^{1}H]-HSQC chemical shift perturbation at a concentration of 100 μM KRAS G12V GMP-PNP. The increasing population of fragment-KRAS complex is visible in the gradually shifting of the KRAS peaks from blue (apo protein) to red (highest ligand concentration measured). A zoom into the amino acids L6, V7, T74, and G75 is presented. The binding curve for T74 (blue) and G75 (orange) is shown for each fragment.

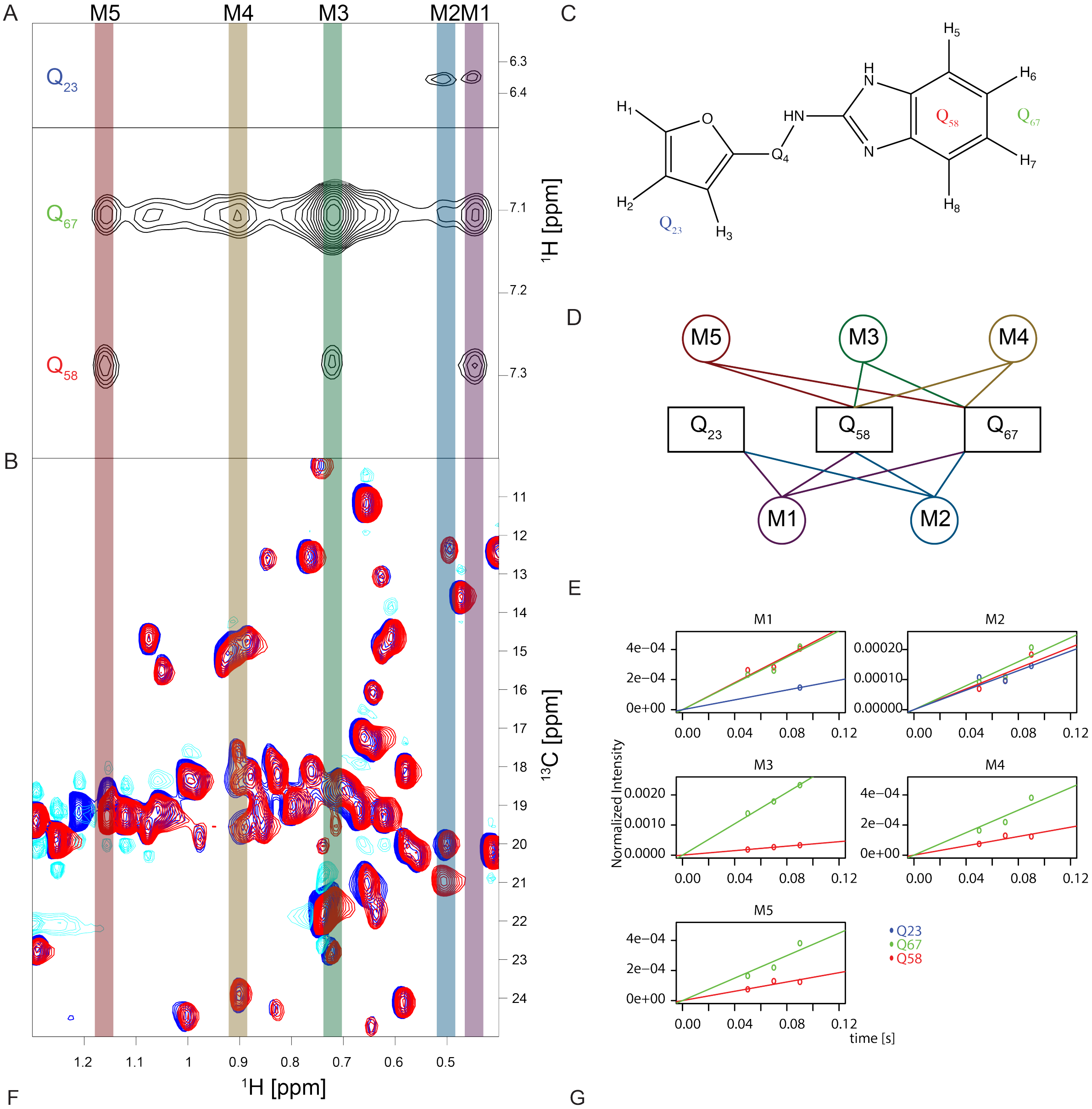

F

Sig. 1	Sig. 2	Dist. [Å]	CYANA Violation [Å]
Q23	Q67	3.0-4.5	upper: 1.12
Q23	H1	2.3-3.4	
Q23	Q58	3.1-4.6	
Q67	Q58	1.5-2.3	upper: 0.44
H1	M5	3.6-24	
H1	M1	3.6-24	
H1	M2	3.6-24	
H1	M3	3.6-24	
H1	M5	3.6-24	

CYANA Target Function = 2.63, $\tau_{c,\ effective}$ = 1.76 ns

G

Sig. 1	Sig. 2	Dist. [Å]	CYANA Violation [Å]
Q23	M1	4.7-5.7	
Q58	M1	4.0-4.8	lower: 0.23
Q67	M1	4.0-4.8	
Q23	M2	4.7-5.7	
Q58	M2	4.6-5.7	
Q67	M2	4.5-5.5	
Q58	M3	4.1-5.0	
Q67	M3	2.9-3.6	
Q58	M4	4.7-5.7	upper: 0.46
Q67	M4	4.1-5.0	upper: 0.24
Q58	M5	3.9-4.8	
Q67	M5	4.1-5.0	upper: 0.25

SI Figure 6. *NMR*^{2} structure determination of fragment **1**. (A) [^{13}C, ^{15}N]-filtered [^{1}H,^{1}H]-NOESY spectra, and (B) the corresponding [^{13}C, 1H]-HSQC spectra of KRAS G12V GMP-PNP in the absence (blue) and presence (red) of fragment **1**. The methyl groups M1-M5 showing cross-peaks to **1** are marked in the spectrum and are summarized as (D) distance restraint network. (E) The NOE build-up curves were generated from the spectra and the (F) intramolecular and **anti-NOE**[1] as well as the (G) intermolecular distance restraints were extracted using Equation 2, where the effective correlation time $\tau_{c,\ effective}$ was extracted by normalization of the distance restraints to a median of 4.5 Å. The restraints were used for *NMR*^{2} structure calculation, with PDB 6XHA as starting structure, resulting in a structure with a CYANA target function of 2.63.

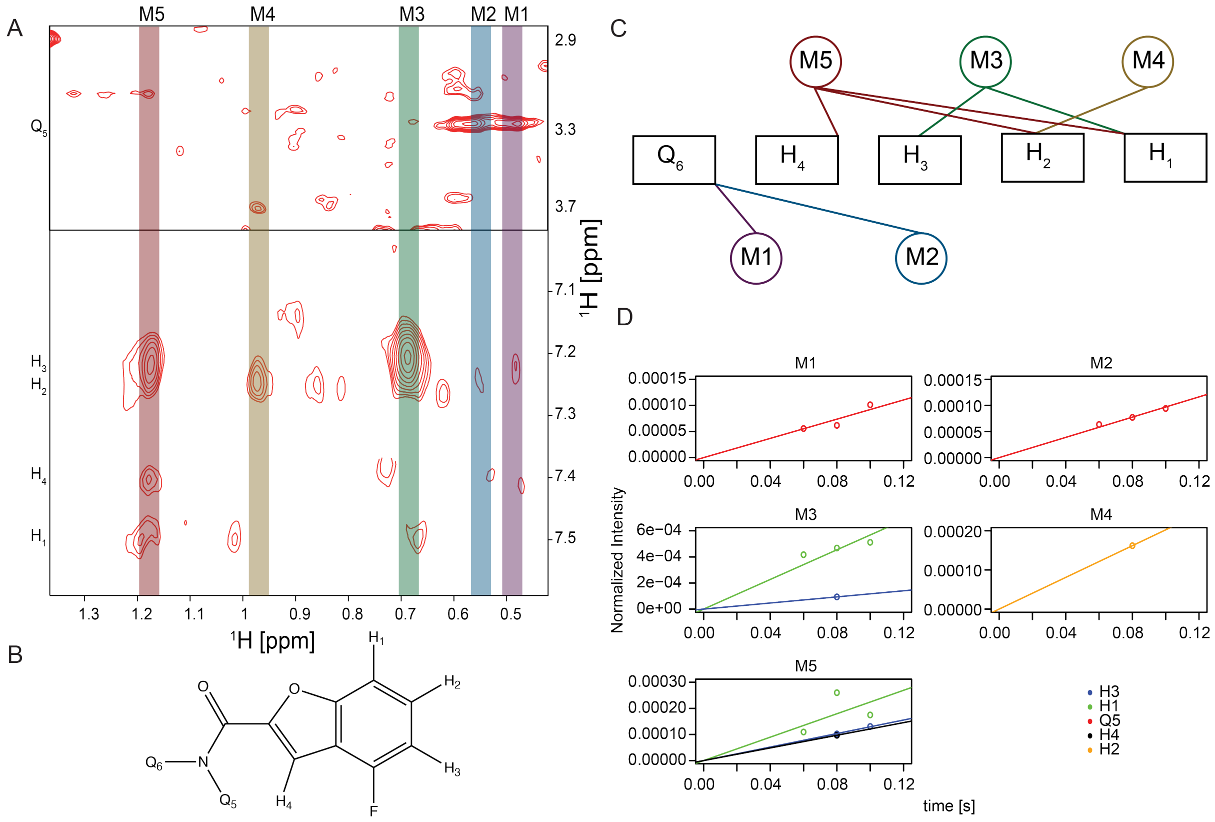

E

Sig. 1	Sig. 2	Dist. [Å]	CYANA Violation [Å]
H2	H3	1.6-2.5	
Q6	Q5	1.2-1.8	upper: 0.70
H4	Q5	3.1-4.8	
H3	Q6	3.3-5.2	upper: 1.92
H2	Q5	3.5-5.5	upper: 1.82
H1	Q5	3.2-5.0	upper: 1.71
H4	Q6	3.4-5.3	
Q6	M4	4.3-30	
Q6	M3	4.3-30	
Q5	M4	4.3-30	
Q5	M3	4.3-30	
Q6	M5	4.3-30	
Q5	M5	4.3-30	

F

Sig. 1	Sig. 2	Dist. [Å]	CYANA Violation [Å]
Q5	M1	4.4-5.4	
Q5	M2	4.3-5.3	
H1	M3	3.3-4.0	
H2	M4	3.9-4.7	upper: 0.23
H3	M4	3.9-4.7	upper: 0.55
H3	M5	4.2-5.1	upper: 0.48
H4	M5	4.2-5.2	
H1	M5	3.8-4.7	

CYANA Target Function = 11.4, $\tau_{c,\ effective}$ = 1.29 ns

SI Figure 7. *N*MR[2] structure determination of fragment **2**. (A) T_1, T_2-filtered [^{1}H,^{1}H]-NOESY spectra of KRAS G12V GMP-PNP in the presence (red) of fragment **2**. The methyl groups M1-M5 showing cross-peaks to **2** are marked in the spectrum and are summarized as (C) distance restraint network. (D) The NOE build-up curves were generated from the spectra and the (E) intramolecular and anti-NOE,[1] as well as the (F) intermolecular distance restraints, were extracted using Equation 2, where the effective correlation time $\tau_{c,effective}$ was extracted by normalization of the distance restraints to a median of 4.5 Å. The restraints were used for *N*MR[2] structure calculation, with PDB 6XHA as starting structure, resulting in a structure with a CYANA target function of 11.4.

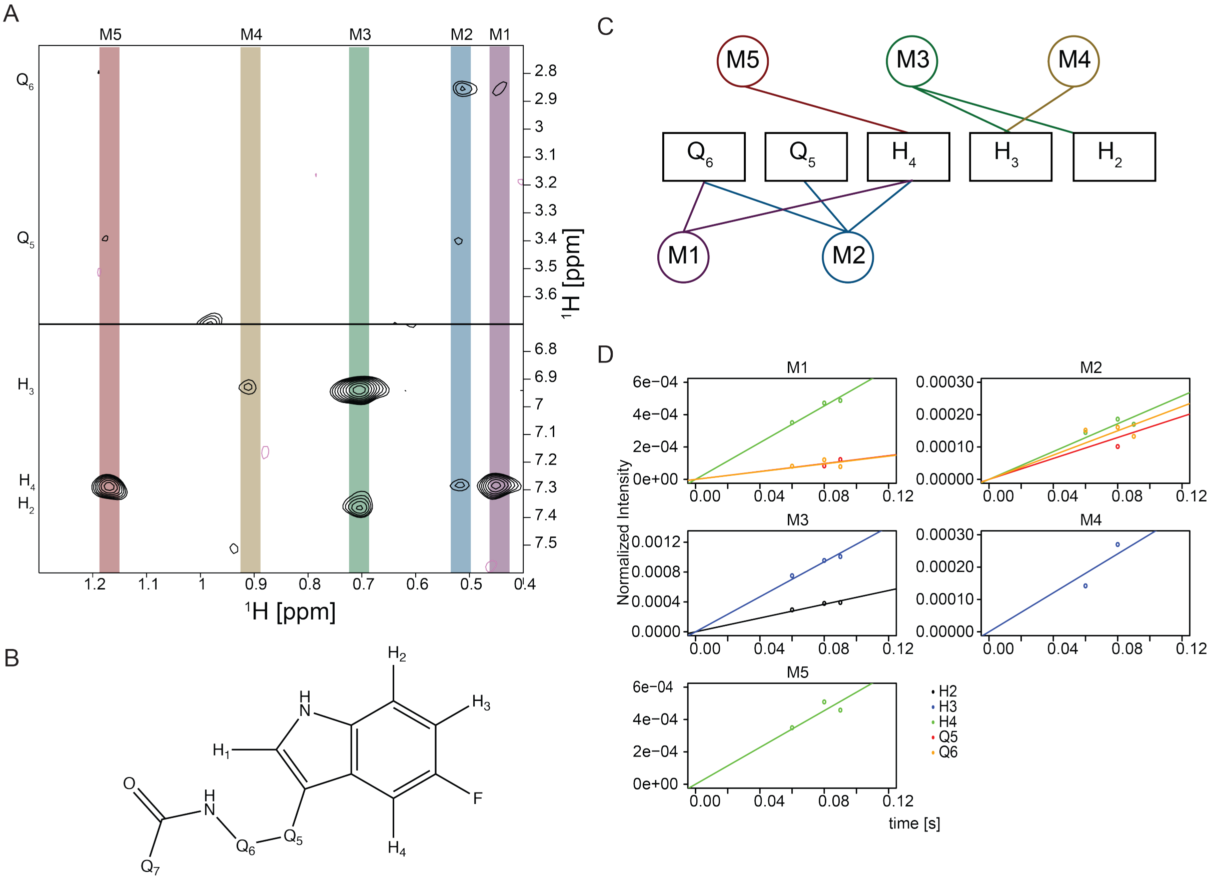

E

Sig. 1	Sig. 2	Dist. [Å]	CYANA Violation [Å]
Q6	Q5	2.2-2.7	
H4	Q6	3.1-3.8	
Q5	H4	2.8-3.4	
Q5	H1	2.6-3.2	
Q7	M1	4.3-30	
Q7	M2	4.3-30	
Q7	M3	4.6-30	
Q7	M4	4.6-30	
Q7	M5	4.3-30	
Q6	M3	4.1-30	
Q6	M4	4.1-30	
Q6	M5	3.8-30	
Q5	M3	4.1-30	
Q5	M4	4.1-30	
Q5	M5	3.8-30	

F

Sig. 1	Sig. 2	Dist. [Å]	CYANA Violation [Å]
H2	M3	3.7-4.4	
H3	M3	3.1-3.8	lower: 0.36
H3	M4	3.9-4.8	upper: 0.40
H4	M1	3.5-4.3	upper: 0.29
H4	M2	4.1-5.1	upper: 0.45
H4	M5	3.5-4.3	upper: 0.43
Q5	M1	4.6-5.6	
Q5	M2	4.4-5.4	
Q6	M1	4.6-5.6	
Q6	M2	4.3-5.2	

CYANA Target Function = 1.14, $\tau_{c,\ effective}$ = 1.79 ns

SI Figure 8. *N*MR^2 structure determination of fragment **3**. (A) T_1, T_2-filtered [^{1}H,^{1}H]-NOESY spectra of KRAS G12V GMP-PNP in the presence of fragment **3**. The methyl groups M1-M5 showing cross-peaks to **3** are marked in the spectrum and are summarized as (C) distance restraint network. (D) The NOE build-up curves were generated from the spectra, and the (E) intramolecular and anti-NOE,[1] as well as the (F) intermolecular distance restraints, were extracted using Equation 2, where the effective correlation time $\tau_{c,effective}$ was extracted by normalization of the distance restraints to a median of 4.5 Å. The restraints were used for *N*MR^2 structure calculation, with PDB 6XHA as starting structure, resulting in a structure with a CYANA target function of 1.14.

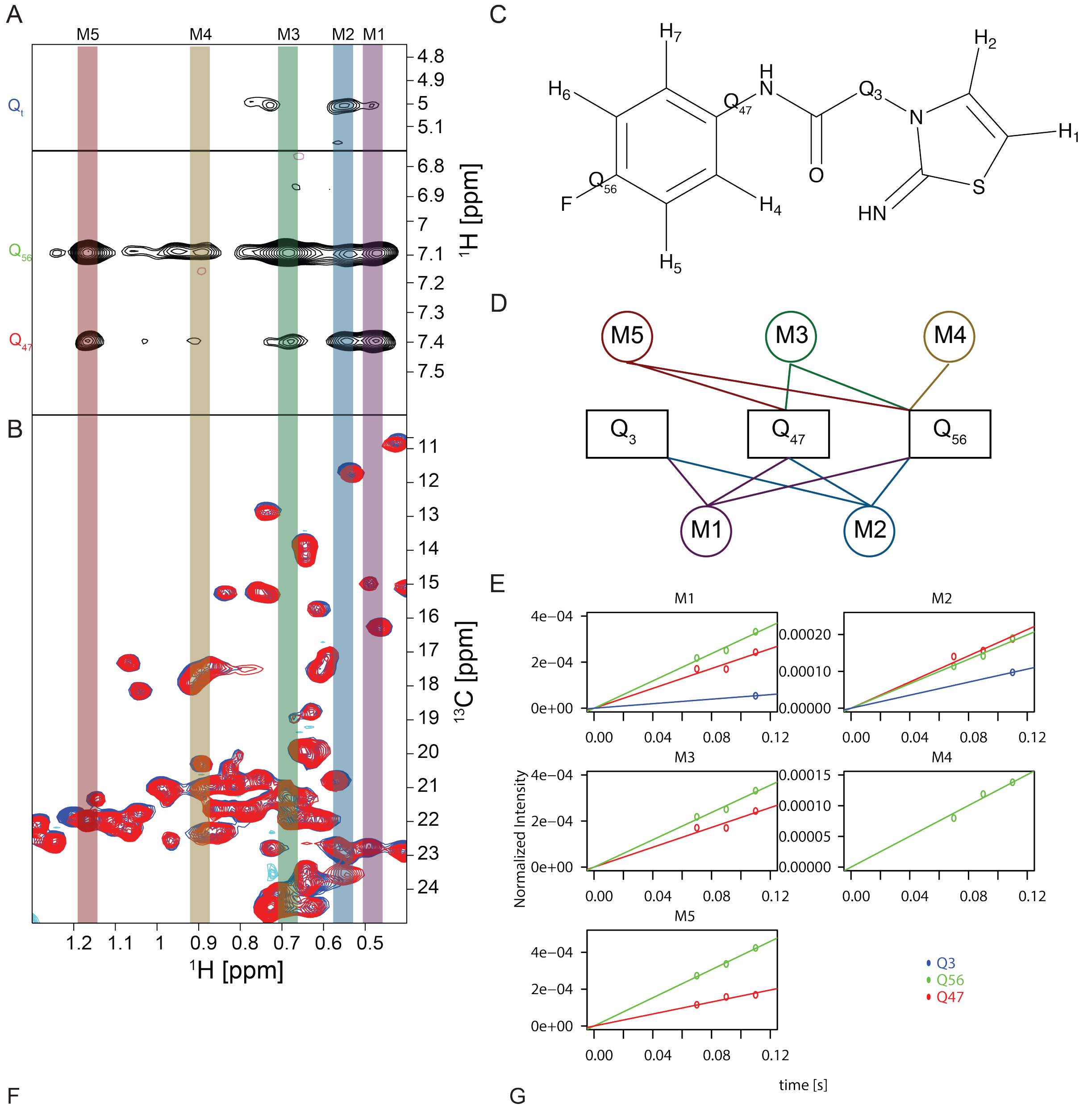

F

Sig. 1	Sig. 2	Dist. [Å]	CYANA Violation [Å]
H1	M3	4.2-25	
H1	M4	4.2-25	
H1	M5	4.2-25	
H2	M3	3.6-25	
H2	M4	3.6-25	
H2	M5	3.6-25	

CYANA Target Function = 1.61, $\tau_{c,\ effective}$ = 1.91 ns

G

Sig. 1	Sig. 2	Dist. [Å]	CYANA Violation [Å]
Q47	M1	3.8-4.7	
Q56	M1	3.6-4.5	lower: 0.26
Q3	M1	4.9-6.0	
Q47	M2	4.0-4.9	
Q56	M2	4.0-4.9	upper: 0.23
Q3	M2	4.5-5.4	
Q47	M3	4.5-5.4	
Q56	M3	3.3-4	
Q56	M4	4.2-5.1	upper: 0.43
Q47	M5	4.0-4.9	upper: 0.35
Q56	M5	3.5-4.3	upper: 0.71

SI Figure 9. *N*MR[2] structure determination of fragment **4**. (A) [^{13}C, ^{15}N]-filtered [^{1}H,^{1}H]-NOESY spectra and (B) the corresponding [^{13}C, 1H]-HSQC spectra of KRAS G12V GMP-PNP in the absence (blue) and the presence (red) of fragment **4**. The methyl groups M1-M5 showing cross-peaks to **4** are marked in the spectrum and are summarized as (D) distance restraint network. (E) The NOE build-up curves were generated from the spectra, and the (F) anti-NOE,[1] as well as the (G) intermolecular distance restraints, were extracted using Equation 2, where the effective correlation time $\tau_{c,\ effective}$ was extracted by normalization of the distance restraints to a

median of 4.5 Å. The restraints were used for *NMR*^{2} structure calculation, with PDB 6XHA as starting structure, resulting in a structure with a CYANA target function of 1.61.

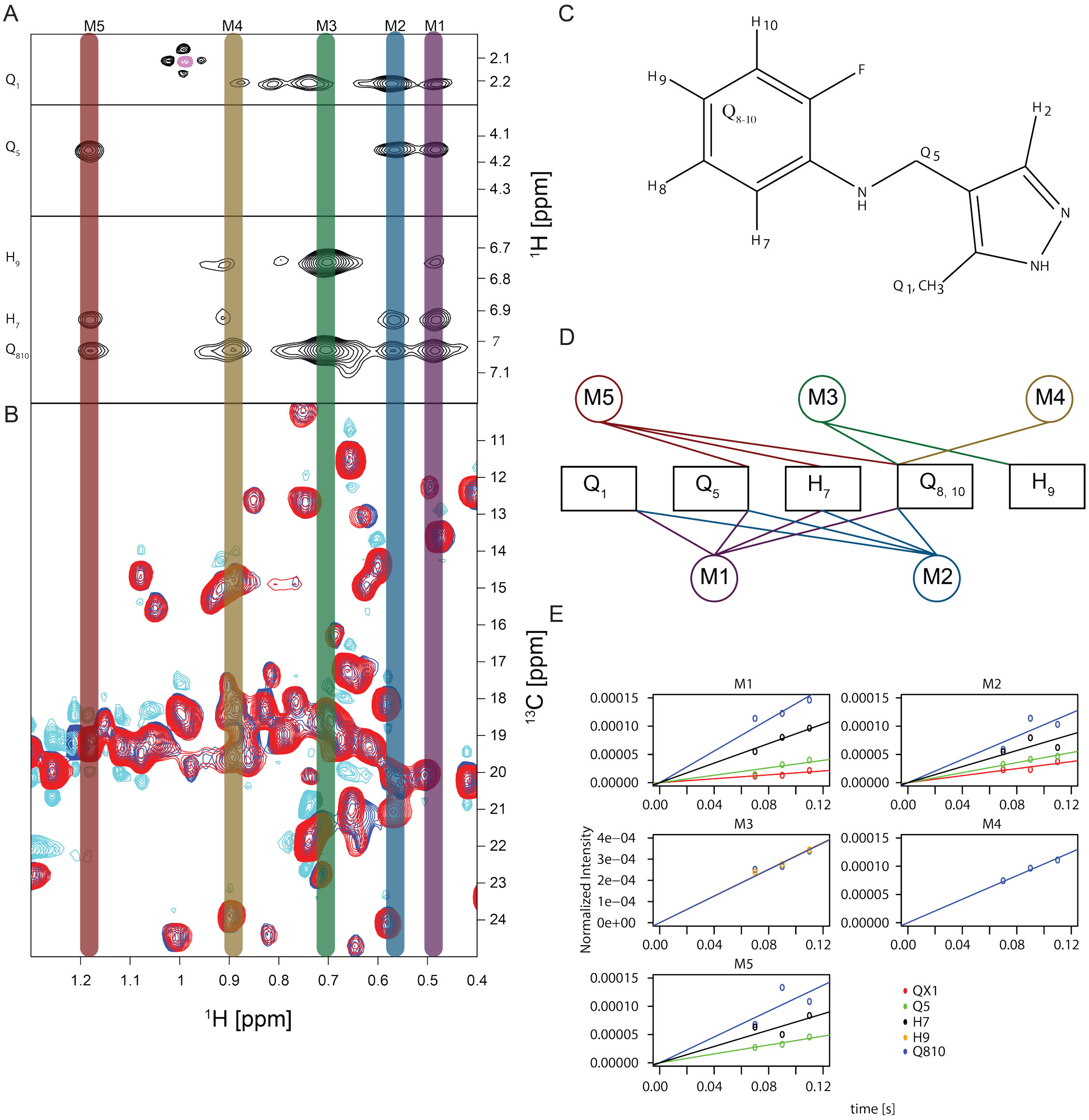

F

Sig. 1	Sig. 2	Dist. [Å]	CYANA Violation [Å]
Q1	H3	4.2-5.2	
Q1	Q5	3.8-4.6	lower: 0.32
Q5	H3	3.5-4.3	lower: 0.54
Q5	H7	3.0-3.6	lower: 0.50
Q1	H7	4.1-5.0	upper: 0.28
H3	H7	3.6-4.4	upper: 0.37

CYANA Target Function = 2.73, $\tau_{c,\ effective}$ = 1.84 ns

G

Sig. 1	Sig. 2	Dist. [Å]	CYANA Violation [Å]
Q1	M1	5.3-6.5	
Q5	M1	4.8-5.9	
Q810	M1	3.8-4.6	
H7	M1	4.1-5.0	
Q1	M2	4.8-5.9	
Q5	M2	4.6-5.6	
Q810	M2	4.1-5.0	
H7	M2	4.2-5.1	upper: 0.26
Q810	M3	3.3-4.1	
H9	M3	3.3-4.0	lower: 0.53
Q810	M4	4.0-4.9	upper: 0.77
Q5	M5	4.7-5.7	
Q810	M5	4.0-4.9	upper: 0.49
H7	M5	4.2-5.1	

SI Figure 10. *NMR*^{2} structure determination of fragment **5**. (A) [^{13}C, ^{15}N]-filtered [^{1}H,^{1}H]-NOESY spectra and (B) the corresponding [^{13}C, 1H]-HSQC spectra of KRAS G12V GMP-PNP in the absence (blue) and the presence (red) of fragment **5**. The methyl groups M1-M5 showing cross-peaks to **5** are marked in the spectrum and are summarized as (D) distance restraint network. (E) The NOE build-up curves were generated from the spectra, and the (F) intramolecular, as well as the (G) intermolecular distance restraints, were extracted using Equation 2, where the effective correlation time $\tau_{c,\ effective}$ was extracted by normalization of the distance restraints to a

median of 4.5 Å. The restraints were used for *NMR*[2] structure calculation, with PDB 6XHA as starting structure, resulting in a structure with a CYANA target function of 2.73.

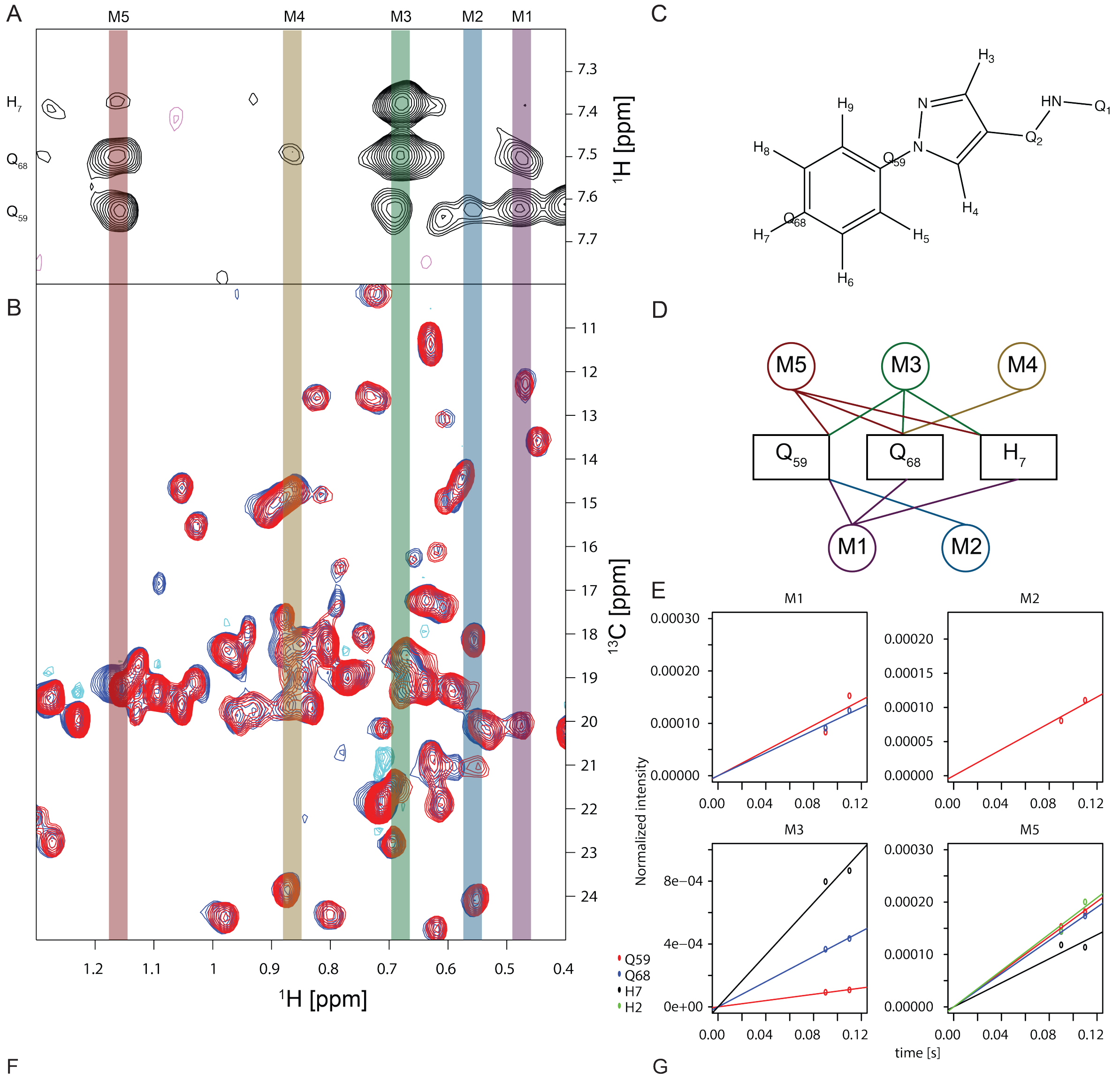

F

Sig. 1	Sig. 2	Dist. [Å]	CYANA Violation [Å]
Q2	M3	4.1-30	
H3	M3	3.6-30	
H4	M3	3.6-30	
Q68	M2	4.3-30	

CYANA Target Function = 3.73, $\tau_{c,\ effective}$ = 1.55 ns

G

Sig. 1	Sig. 2	Dist. [Å]	CYANA Violation [Å]
Q59	M1	4.1-5.3	lower: 0.30
Q68	M1	4.2-5.4	lower: 0.55
Q59	M2	4.2-5.5	
Q59	M3	4.2-5.5	upper: 0.51
H7	M3	2.9-3.8	upper: 0.40
Q68	M3	3.4-4.3	
Q2	M5	0.0-5.1	upper: 0.84
Q59	M5	3.9-5.0	upper: 0.30
H7	M5	0.0-5.3	upper: 0.71
Q68	M5	3.9-5.1	upper: 0.63

SI Figure 11. *NMR*[2] structure determination of fragment **9**. (A) [T_1, T_2]-filtered [^1H,^1H]-NOESY spectra and (B) the corresponding [^{13}C, 1H]-HSQC spectra of KRAS G12V GMP-PNP in the absence (blue) and the presence (red) of fragment **9**. The methyl groups M1-M5 showing cross-peaks to **9** are marked in the spectrum and are summarized as (D) distance restraint network. (E) The NOE build-up curves were generated from the spectra, and the (F) anti-NOE,[1] as well as the (G) intermolecular distance restraints, were

extracted using Equation 2, where the effective correlation time $\tau_{c,\,effective}$ was extracted by normalization of the distance restraints to a median of 4.5 Å. The restraints were used for *NMR*² structure calculation, with PDB 6XHA as starting structure, resulting in a structure with a CYANA target function of 3.73.

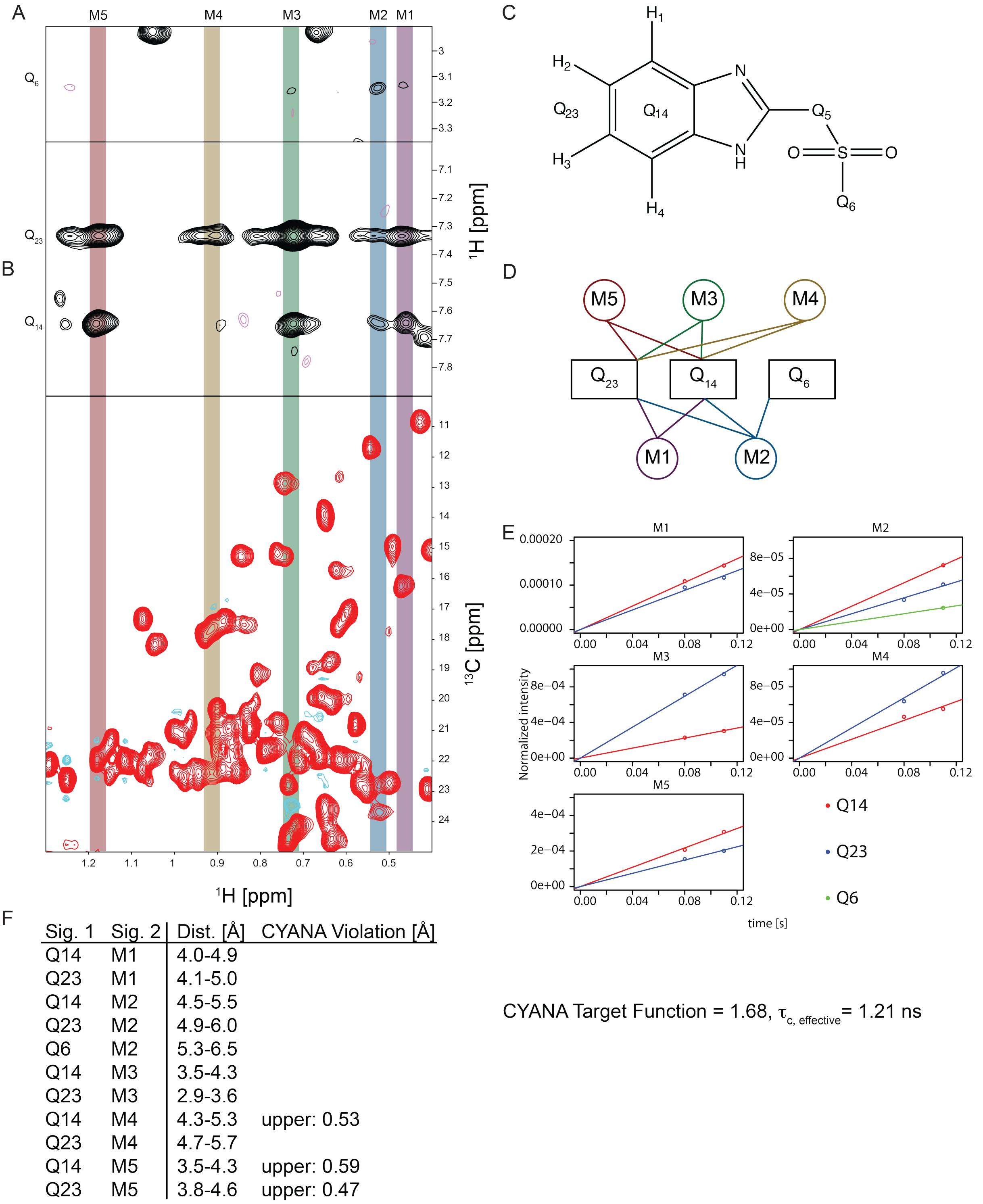

F

Sig. 1	Sig. 2	Dist. [Å]	CYANA Violation [Å]
Q14	M1	4.0-4.9	
Q23	M1	4.1-5.0	
Q14	M2	4.5-5.5	
Q23	M2	4.9-6.0	
Q6	M2	5.3-6.5	
Q14	M3	3.5-4.3	
Q23	M3	2.9-3.6	
Q14	M4	4.3-5.3	upper: 0.53
Q23	M4	4.7-5.7	
Q14	M5	3.5-4.3	upper: 0.59
Q23	M5	3.8-4.6	upper: 0.47

CYANA Target Function = 1.68, $\tau_{c,\,effective}$ = 1.21 ns

SI Figure 12. *NMR*² structure determination of fragment **11**. (A) [T_1, T_2]-filtered [^{1}H,^{1}H]-NOESY spectra and (B) the corresponding [^{13}C, 1H]-HSQC spectra of KRAS G12V GMP-PNP in the presence (red) of fragment **11**. The methyl groups M1-M5 showing cross-peaks to **11** are marked in the spectrum and are summarized as (D) distance restraint network. (E) The NOE build-up curves were generated from the spectra, and the (F) intermolecular distance restraints were extracted using Equation 2, where the effective

correlation time $\tau_{c,\ effective}$ was extracted by normalization of the distance restraints to a median of 4.5 Å. The restraints were used for *NMR*[2] structure calculation, with PDB 6XHA as starting structure, resulting in a structure with a CYANA target function of 1.68.

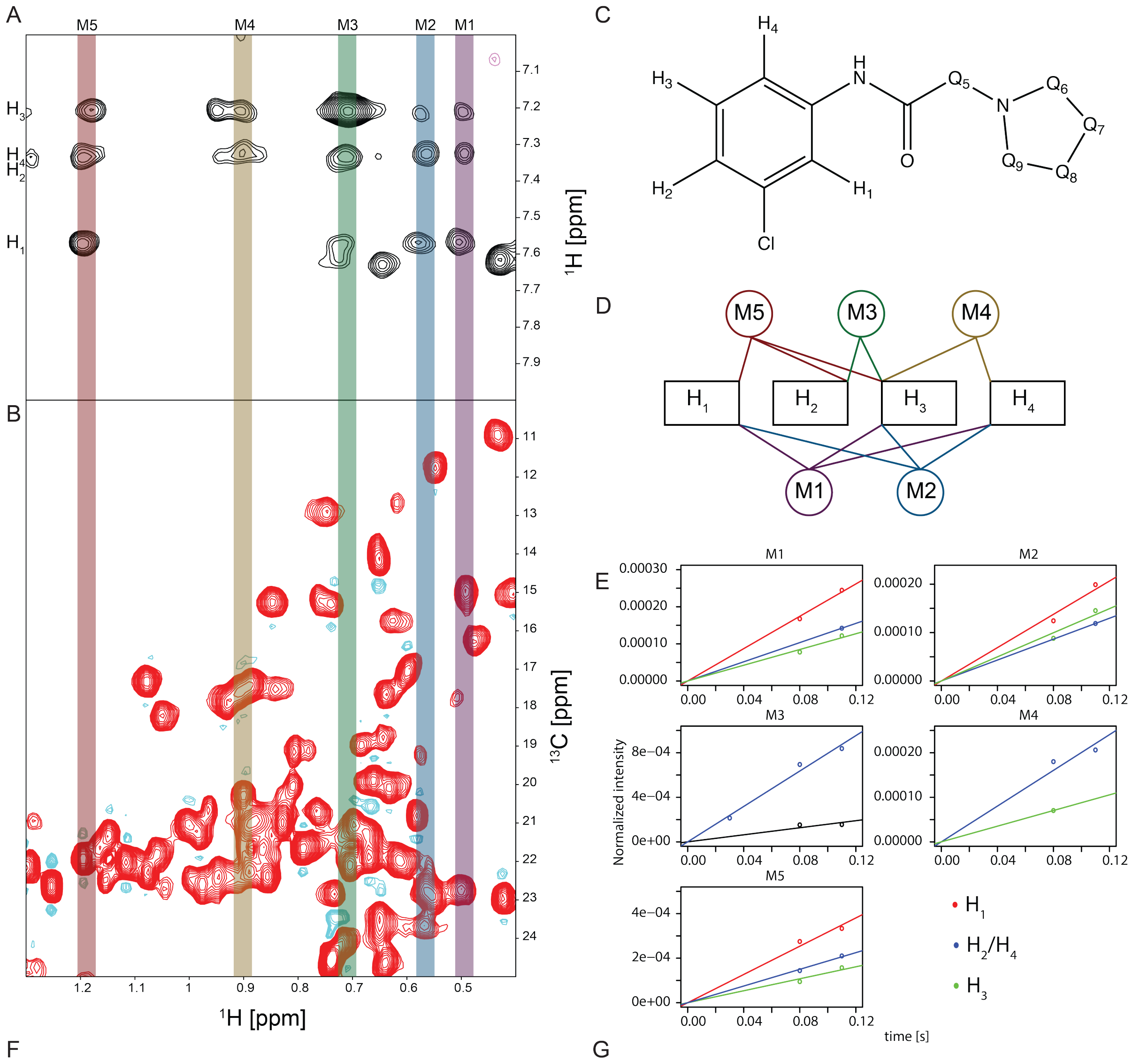

F

Sig. 1	Sig. 2	Dist. [Å]	CYANA Violation [Å]
Q69	H1	3.3-7.2	
Q69	H4	3.2-7.1	
Q69	H3	3.4-7.6	
Q78	H4	3.4-7.6	
Q69	M3	4.1-30	
Q5	M3	4.1-30	
Q78	M3	4.1-30	
Q69	M4	4.1-30	
Q5	M4	4.1-30	
Q78	M4	4.1-30	
Q69	M5	3.4-30	
Q5	M5	3.4-30	
Q78	M5	3.4-30	

G

Sig. 1	Sig. 2	Dist. [Å]	CYANA Violation [Å]
H1	M1	3.8-4.7	upper: 0.70
H3	M1	4.2-5.1	
H4	M1	4.3-5.3	
H1	M2	4.0-4.9	
H3	M2	4.2-5.2	upper: 0.40
H4	M2	4.3-5.3	
H2	M3	4.0-4.9	
H3	M3	3.1-3.8	
H3	M4	3.9-4.8	upper: 0.88
H1	M5	3.6-4.4	upper: 1.18
H3	M5	3.9-4.8	upper: 1.43

CYANA Target Function = 6.33, $\tau_{c,\ effective}$ = 1.20 ns

SI Figure 13. *NMR*[2] structure determination of fragment **12**. (A) [T_1, T_2]-filtered [^{1}H,^{1}H]-NOESY spectra and (B) the corresponding [^{13}C, 1H]-HSQC spectra of KRAS G12V GMP-PNP in the presence (red) of fragment **12**. The methyl groups M1-M5 showing cross-peaks to **12** are marked in the spectrum and are summarized as (D) distance restraint network. (E) The NOE build-up curves were

generated from the spectra, and the (F) intramolecular and anti-NOE,[1] as well as the (G) intermolecular distance restraints, were extracted using Equation 2, where the effective correlation time $\tau_{c,\ effective}$ was extracted by normalization of the distance restraints to a median of 4.5 Å. The restraints were used for *NMR*[2] structure calculation, with PDB 6XHA as starting structure, resulting in a structure with a CYANA target function of 6.33.

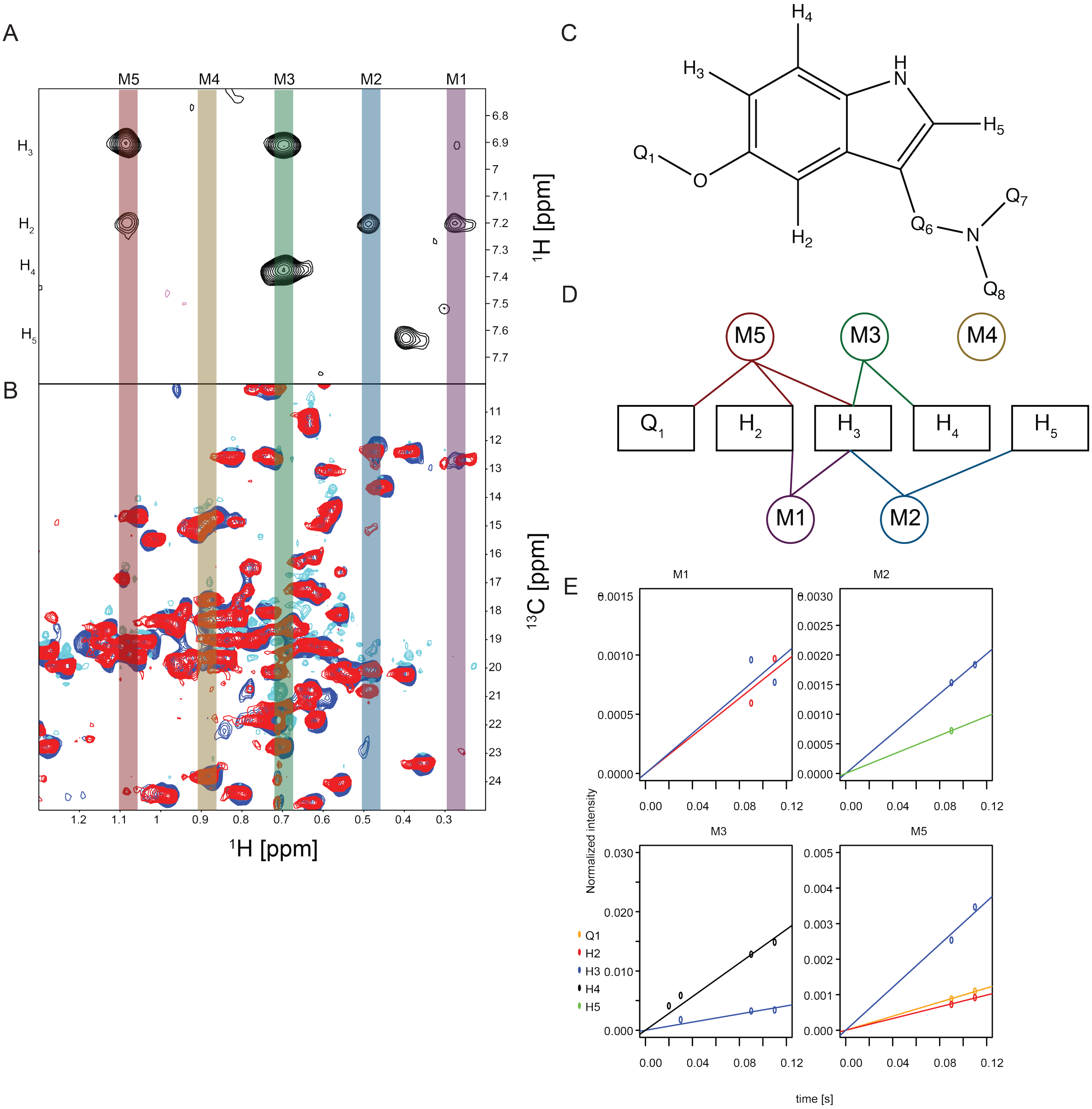

F

Sig. 1	Sig. 2	Dist. [Å]	CYANA Violation [Å]
Q78	M5	3.8-9	
Q78	M2	3.6-9	
Q78	M3	5.0-30	
Q78	M4	5.0-30	
Q6	M3	4.8-30	
Q6	M4	4.8-30	

CYANA Target Function = 1.19, $\tau_{c,\ effective}$ = 2.15 ns

G

Sig. 1	Sig. 2	Dist. [Å]	CYANA Violation [Å]
H2	M1	4.1-5.4	upper: 0.35
H3	M1	4.1-5.5	
H3	M2	3.6-4.8	
H5	M2	6.1-8.2	
H3	M3	3.2-4.3	
H4	M3	2.5-3.4	
H1	M5	4.0-5.3	
H2	M5	4.1-5.4	upper: 0.47
H3	M5	3.3-4.4	

SI Figure 14. *NMR*[2] structure determination of fragment **13**. (A) [T_1, T_2]-filtered [^{1}H,^{1}H]-NOESY spectra and (B) the corresponding [^{13}C, 1H]-HSQC spectra of KRAS G12V GMP-PNP in the absence (blue) and the presence (red) of fragment **13**. The methyl groups M1-M5 showing cross-peaks to **13** are marked in the spectrum and are summarized as (D) distance restraint network. (E) The NOE

build-up curves were generated from the spectra, and the (F) anti-NOE,[1] as well as the (G) intermolecular distance restraints, were extracted using Equation 2, where the effective correlation time $\tau_{c,\ effective}$ was extracted by normalization of the distance restraints to a median of 4.5 Å. The restraints were used for *N*MR[2] structure calculation, with PDB 6XHA as starting structure, resulting in a structure with a CYANA target function of 1.19.

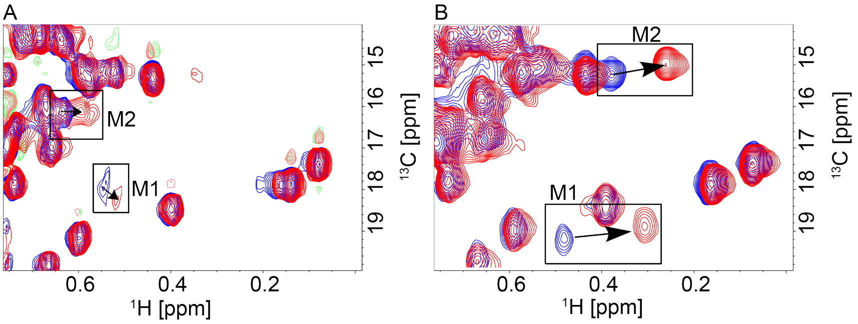

SI Figure 15. A zoom into the methyl region of M1 and M2 of the [^{13}C, ^{1}H]-HSQC spectra of KRAS G12V is shown in the (A) GMP-PNP and (B) GDP state in the absence (blue) of a ligand or in the presence (red) of compound **9** (A) or indole (B).

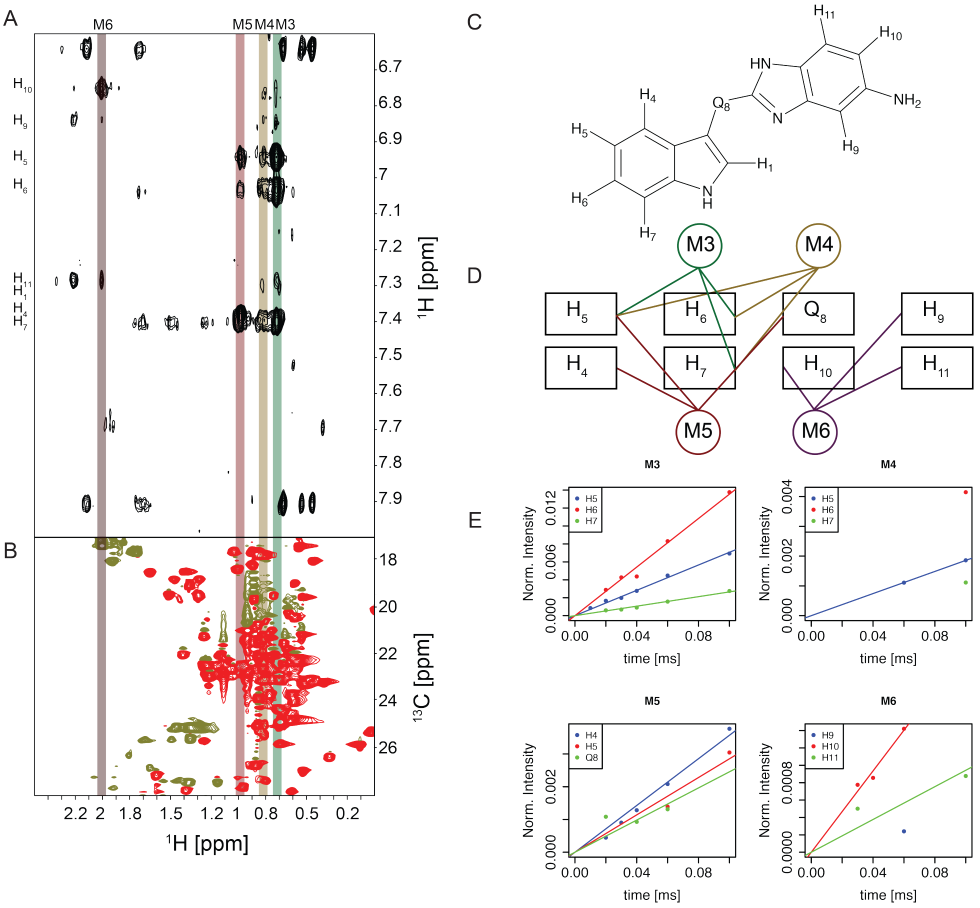

F

Sig. 1	Sig. 2	Dist. [Å]	CYANA Violation [Å]
H5	H4	3.3-4.0	
H10	H11	3.0-3.6	
H4	Q8	3.9-4.7	
H1	Q8	4.1-5.0	
H5	Q8	3.6-10	
H6	H7	4.5-5.5	
H9	Q8	3.7-4.5	Upper: 0.60
H10	Q8	3.8-4.7	Upper: 1.08
H6	Q8	4.3-10	

CYANA Target Function = 3.98, $\tau_{c,\ effective}$ = 3.5 ns

G

Sig. 1	Sig. 2	Dist. [Å]
H5	M3	3.3-4.0
H6	M3	3.0-3.6
H7	M3	3.9-4.7
H5	M4	4.1-10
H6	M4	3.6-10
H7	M4	4.5-10
H4	M5	3.7-4.5
H5	M5	3.8-4.7
Q8	M5	4.3-5.2
H9	M6	5.8-7.0
H10	M6	4.3-5.2
H11	M6	4.6-5.6

H

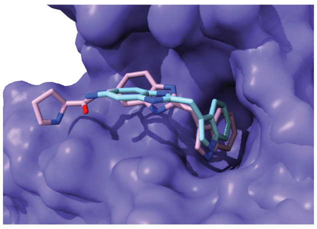

SI Figure 16. *NMR*^{2} structure determination of fragment PDB 4EPY analogue. (A) [^{13}C, ^{15}N]-filtered [^{1}H,^{1}H]-NOESY spectra and (B) the corresponding [^{13}C, 1H]-HSQC spectra of KRAS G12V GDP in the presence (red) of the ligand depicted in (C). The methyl

groups M3-M6 that show cross-peaks to the ligand are marked in the spectrum and are summarized as (D) a distance restraint network. (E) The NOE build-up curves were generated from the spectra, and the (F) anti-NOE and G) intermolecular distance restraints, were extracted using Equation 2, where the effective correlation time $\tau_{c,\ effective}$ was extracted by normalization of the distance restraints to a median of 4.5 Å. The restraints were used for *N*MR^2 structure calculation, with PDB 8G4F as starting structure, resulting in a structure with a CYANA target function of 3.98. (H) Overlay of the X-ray structure of 4EPY (pink) and the *N*MR^2 structure (9QLW, light blue).

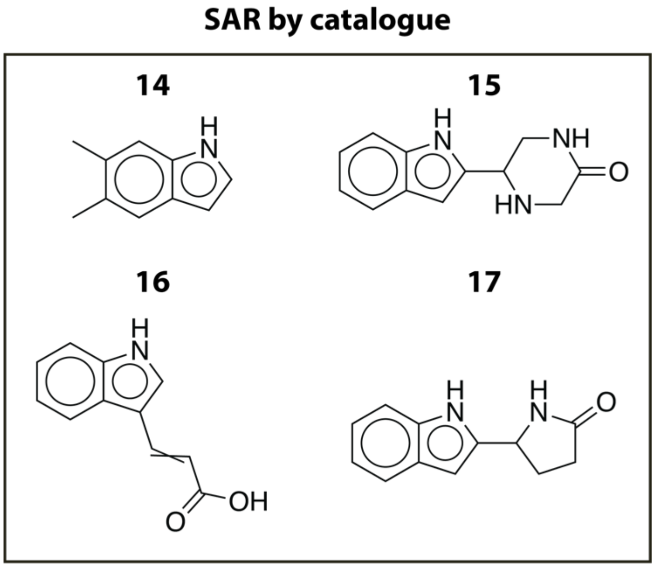

SI Figure 17. 2D representation of the fragments generated by the findings in the pharmacophore generated from the *N*MR^2 structures of the hits.

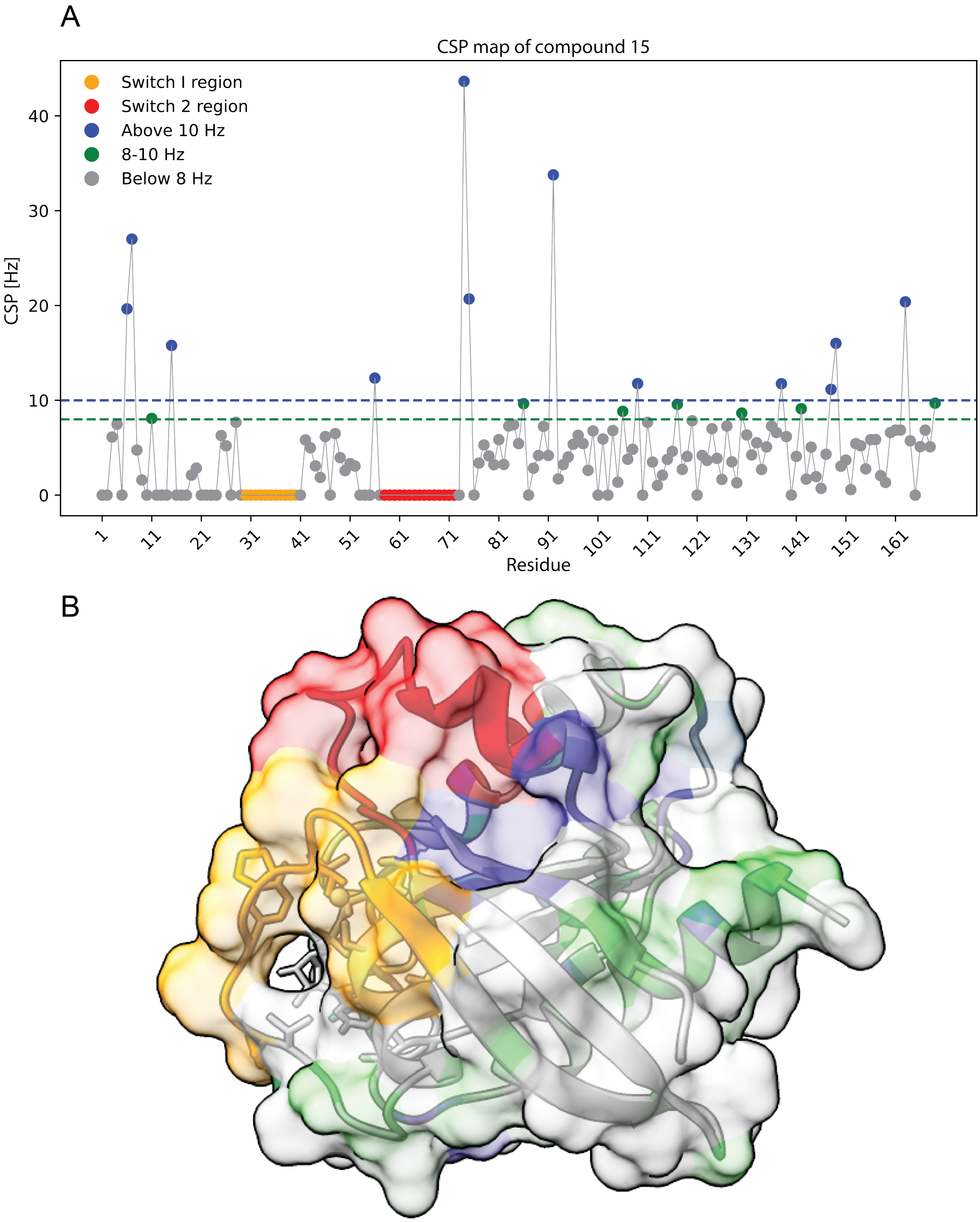

SI Figure 18. (A) Chemical shift perturbation map of KRAS G12V GMP-PNP upon addition of 1 mM of compound **15**. Shifts higher than 10 Hz are shown in blue and higher than 8 in green. Switch I and II regions are shown in orange and red, respectively. (B) Color coding mapped on crystal structure 6XHA.

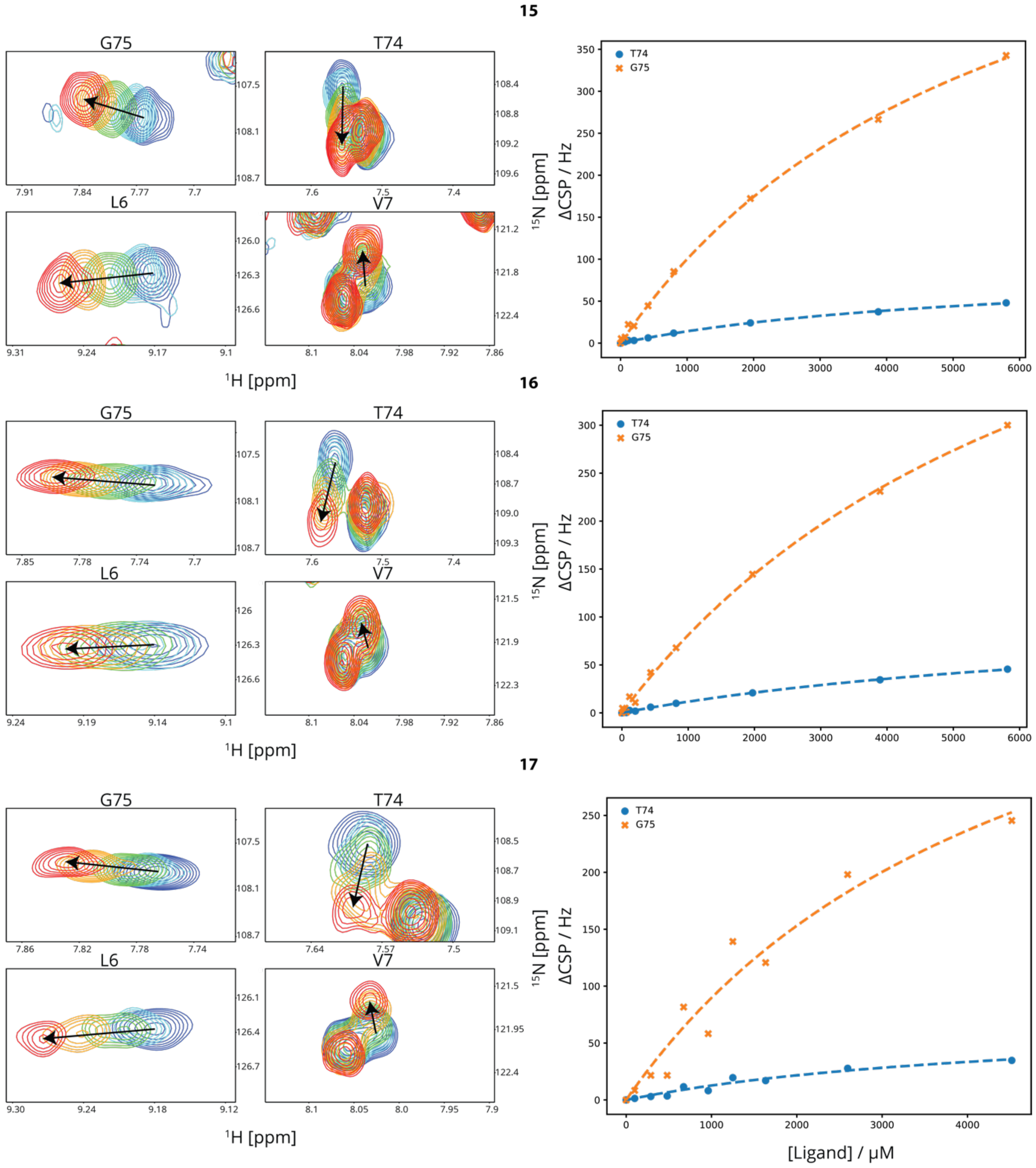

SI Figure 19. Ligand titrated [^{15}N, ^{1}H]-HSQC chemical shift perturbation at a concentration of 100 µM KRAS G12V GMP-PNP and fragments **15**, **16** and **17**. The increasing population of fragment-KRAS complex is visible in the gradually shifting of the KRAS peaks from blue (apo protein) to red (highest ligand concentration measured). A zoom into the amino acids L6, V7, T74, and G75 is presented. The binding curve for T74 (blue) and G75 (orange) is shown for each fragment.

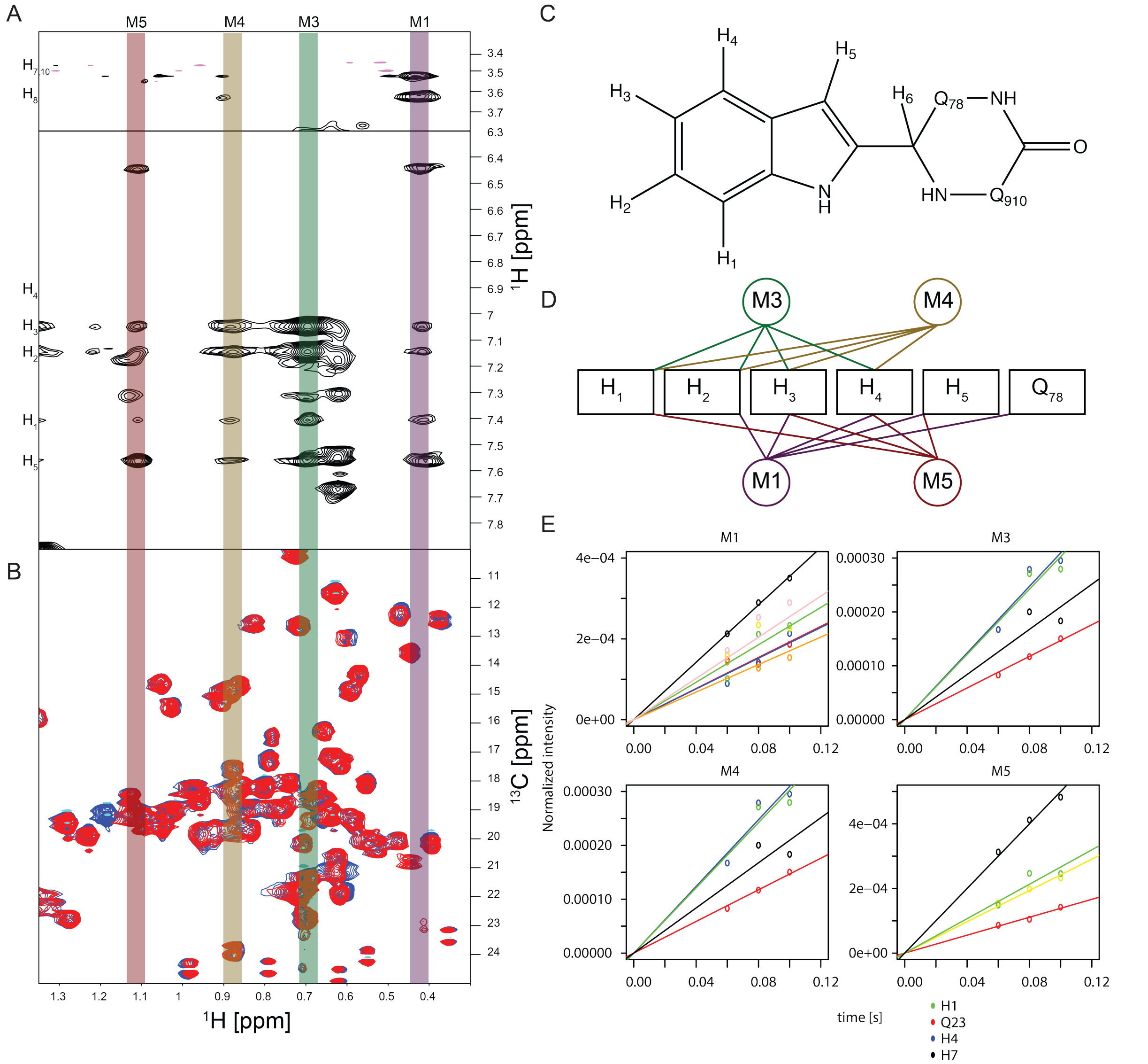

F

Sig. 1	Sig. 2	Dist. [Å]	CYANA Violation [Å]
H3	H5	2.3-3.6	upper: 0.99
H4	H5	1.7-2.6	upper: 1.60
H1	M4	3.6-7.2	
H3	M4	3.7-7.2	
H4	M4	3.7-7.2	
H1	M5	4.2-30	
H2	M5	4.2-30	
H5	M3	4.2-30	
H6	M3	4.2-30	
H7	M4	3.6-30	
H9	M4	3.6-30	

CYANA Target Function = 5.98, $\tau_{c,\ effective}$ = 1.32 ns

G

Sig. 1	Sig. 2	Dist. [Å]	CYANA Violation [Å]
H1	M1	4.2-5.2	lower: 0.44
H4	M1	3.9-4.7	
H5	M1	4.1-5.0	
H8	M1	4.4-5.3	upper: 0.61
H1	M3	4.0-4.9	
H2	M3	3.2-3.9	lower: 0.36
H3	M3	3.1-3.7	
H4	M3	3.6-4.5	
H2	M4	3.9-4.8	upper: 0.73
H3	M5	4.0-4.9	upper: 0.99
H4	M5	3.6-4.5	
H5	M5	4.1-5.0	

SI Figure 20. *N*MR[2] structure determination of fragment **15**. (A) [^{13}C, ^{15}N]-filtered [^{1}H,^{1}H]-NOESY spectra, and (B) the corresponding [^{13}C, 1H]-HSQC spectra of KRAS G12V GMP-PNP in the absence (blue) and presence (red) of fragment **15**. The methyl groups M1-M5 showing cross-peaks to **15** are marked in the spectrum and are summarized as (D) distance restraint network. (E) The NOE build-up curves were generated from the spectra, and the (F) intramolecular and anti-NOE,[1] as well as the (G) intermolecular distance restraints, were extracted using Equation 2, where the effective correlation time $\tau_{c,\ effective}$ was extracted by

normalization of the distance restraints to a median of 4.5 Å. The restraints were used for *NMR*^{2} structure calculation, with PDB 6XHA as starting structure, resulting in a structure with a CYANA target function of 5.98.

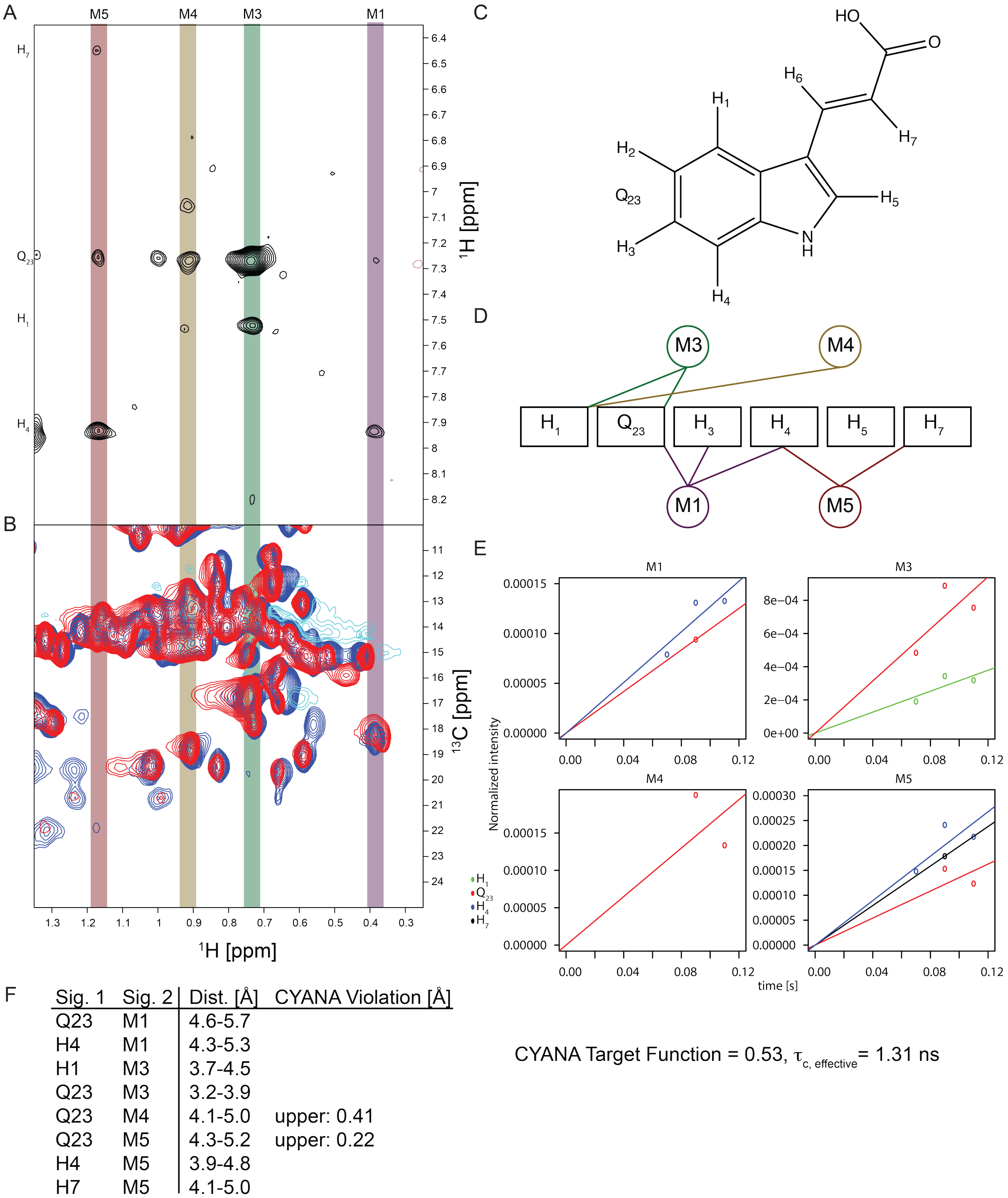

F

Sig. 1	Sig. 2	Dist. [Å]	CYANA Violation [Å]
Q23	M1	4.6-5.7	
H4	M1	4.3-5.3	
H1	M3	3.7-4.5	
Q23	M3	3.2-3.9	
Q23	M4	4.1-5.0	upper: 0.41
Q23	M5	4.3-5.2	upper: 0.22
H4	M5	3.9-4.8	
H7	M5	4.1-5.0	

CYANA Target Function = 0.53, $\tau_{c,\ effective}$ = 1.31 ns

SI Figure 21. *NMR*^{2} structure determination of fragment **16**. (A) [^{13}C, ^{15}N]-filtered [^{1}H,^{1}H]-NOESY spectra, and (B) the corresponding [^{13}C, 1H]-HSQC spectra of KRAS G12V GMP-PNP in the absence (blue) and presence (red) of fragment **16**. The methyl groups M1-M5 showing cross-peaks to **16** are marked in the spectrum and are summarized as (D) distance restraint network. (E) The NOE build-up curves were generated from the spectra, and the (F) intermolecular distance restraints were extracted using Equation 2, where the effective correlation time $\tau_{c,\ effective}$ was extracted by normalization of the distance restraints to a median of 4.5 Å. The restraints were used for *NMR*^{2} structure calculation, with PDB 6XHA as starting structure, resulting in a structure with a CYANA target function of 0.53.

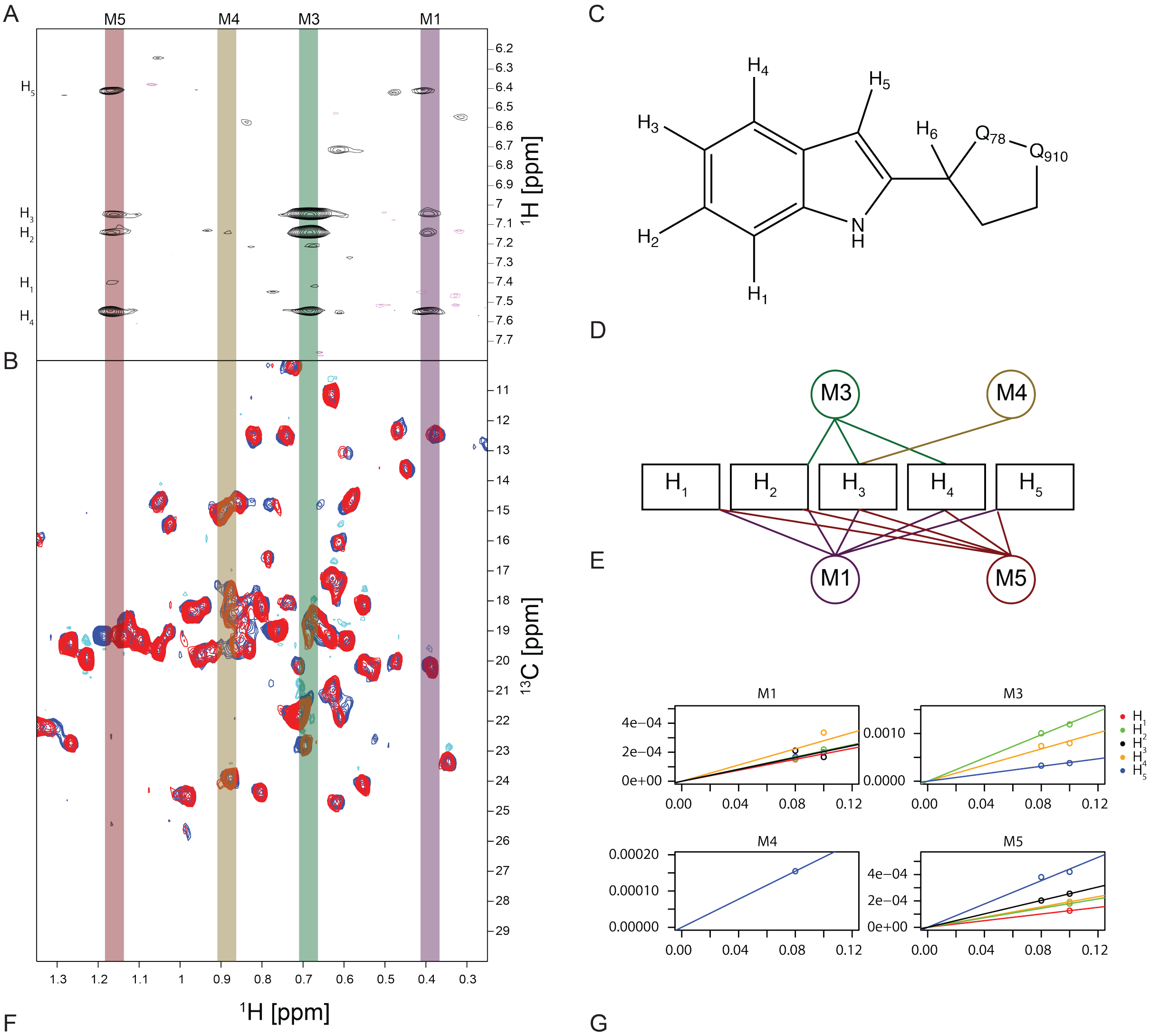

F

Sig. 1	Sig. 2	Dist. [Å]	CYANA Violation [Å]
H4	H5	2.4-2.9	
H5	H6	2.5-3.1	upper: 0.79
H5	H7	2.5-3.1	
H5	H9	2.7-3.3	upper: 1.14

CYANA Target Function = 4.32, $\tau_{c,\ effective}$ = 1.31 ns

G

Sig. 1	Sig. 2	Dist. [Å]	CYANA Violation [Å]
H1	M1	4.1-5.1	
H2	M1	3.9-4.7	
H3	M1	4.1-5.0	
H4	M1	4.1-5.0	
H5	M1	4.1-5.0	
H2	M3	3.2-3.9	
H3	M3	3.0-3.7	
H4	M3	3.7-4.5	
H3	M4	4.1-5.0	
H1	M5	4.4-5.4	
H2	M5	4.1-5.0	
H3	M5	4.2-5.1	
H4	M5	3.6-4.4	upper: 0.40
H5	M5	3.9-4.8	

SI Figure 22. *N*MR^2 structure determination of fragment **17**. (A) [T_1, T_2]-filtered [^{1}H,^{1}H]-NOESY spectra and (B) the corresponding [^{13}C, 1H]-HSQC spectra of KRAS G12V GMP-PNP in the absence (blue) and the presence (red) of fragment **17**. The methyl groups M1-M5 showing cross-peaks to **17** are marked in the spectrum and are summarized as (D) distance restraint network. (E) The NOE build-up curves were generated from the spectra, and the (F) intramolecular, as well as the (G) intermolecular distance restraints, were extracted using Equation 2, where the effective correlation time $\tau_{c,\ effective}$ was extracted by normalization of the distance restraints to a median of 4.5 Å. The restraints were used for *N*MR^2 structure calculation, with PDB 6XHA as starting structure, resulting in a structure with a CYANA target function of 4.32

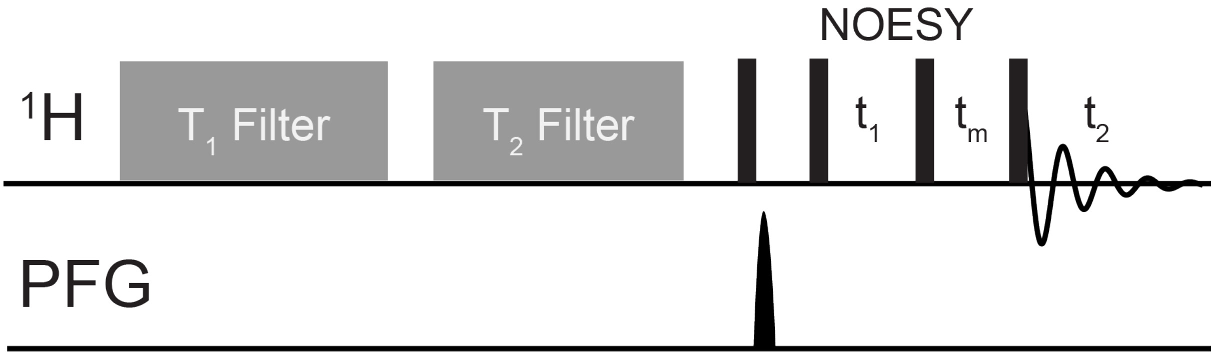

SI Figure 23. T_1, T_2 - relaxation filtered NOESY pulse sequence used to derive protein-ligand NOE cross-peaks without the use of isotope labeling.

REFERENCES

(1) Torres, F.; Walser, R.; Kaderli, J.; Rossi, E.; Bobby, R.; Packer, M. J.; Sarda, S.; Walker, G.; Hitchin, J. R.; Milbradt, A. G.; Orts, J. NMR Molecular Replacement Provides New Insights into Binding Modes to Bromodomains of BRD4 and TRIM24. *J. Med. Chem.* **2022**, *65* (7), 5565–5574. https://doi.org/10.1021/acs.jmedchem.1c01703.
